# Supplementary material for: Mothers at risk of postpartum depression in Sri Lanka: A population-based study using a validated screening tool
Source: PLoS One. 2022 May 25;17(5):e0268748. doi: 10.1371/journal.pone.0268748 (PMC9132337; doi:10.1371/journal.pone.0268748)
Supplement: Attachment — Submitted filename: PONE-D-21-20712_R1.pdf [file pone.0268748.s003.pdf]

PLOS ONE

Risk of postpartum depression in Sri Lanka: A population-based study using a validated screening tool

--Manuscript Draft--

**Manuscript Number:**	PONE-D-21-20712R1
**Article Type:**	Research Article
**Full Title:**	Risk of postpartum depression in Sri Lanka: A population-based study using a validated screening tool
**Short Title:**	Postpartum depression in Sri Lanka
**Corresponding Author:**	Therese Røysted Solås<br>University of Bergen Faculty of Medicine and Dentistry: Universitetet i Bergen Det medisinsk-odontologiske fakultet<br>Bergen, NORWAY
**Keywords:**	postpartum depression; screening; Sri Lanka; cross-sectional study
**Abstract:**	Background<br>Postpartum depression is an important public health concern. The prevalence of postpartum depression is estimated to be 18% worldwide. The purpose of this study was to estimate the prevalence of postpartum depression in Sri Lanka and to investigate its associated risk factors.<br><br>Methods<br>This was a cross-sectional study conducted among 975 mothers in Galle district, Sri Lanka. The prevalence of mothers at risk of postpartum depression was assessed using the Edinburgh Postpartum Depression scale (EPDS) which has been validated for screening for postpartum depression in Sri Lanka with a cut-off score 9 or more. Data from routine records on pregnancy, delivery, postnatal care was collected to investigate possible predictors of postpartum depression. Crosstabulation and multivariable logistic regression was performed to identify risk factors for postpartum depression, defined as EPDS score 9 or more. Prevalence estimates using different cut-offs for postpartum depression was compared to assess the impact of using unvalidated cut-offs for screening.<br><br>Results<br>The prevalence of mothers with EPDS score 9 or more was found to be 9.4% (95%CI: 7.8-11.4); EPDS score 10 or more was 5.6% (95%CI: 4.4-7.3). Postpartum depression (EPDS 9 or more) was associated with the following risk factors: Former history of mental illness (aOR 32.9, 95%CI: 7.9-136.2), high maternal age 30-39 (aOR 2.2, 95%CI: 1.3-3.8), BMI 25.0-29.9 (aOR 2.6, 95%CI: 1.5-4.5), hypertension (aOR 3.6, 95%CI: 1.2-10.9) and newborn death (aOR 28.9, 95%CI: 4.5-185.1). One in five women reported thoughts of self-harm.<br><br>Conclusion<br>A significant proportion of Sri Lankan mothers experience symptoms of postpartum depression, highest risk among mothers who reported former history of mental illness and newborn death. Using a higher cut-off for screening had a substantial impact on the prevalence rate estimates. Mothers at increased risk should be identified in antenatal care and is an important target of referral.
**Order of Authors:**	Therese Røysted Solås
	Sven Gudmund Hinderaker
	Lasantha Ubesekara
	Vijitha De Silva
**Opposed Reviewers:**	
**Response to Reviewers:**	EDITOR

Powered by Editorial Manager® and ProduXion Manager® from Aries Systems Corporation

	Additional Editor Comments:<br><br>Comment 1: The title of the current paper and that by Fan et al., 2020 are quite similar. Could you please come out clearly as to what differs between your paper and the earlier published one, more so when the study population seems to be the same<br>Response: Thank you for pointing this out. In the revised version we have chosen to highlight that this study uses the validated cut-off for screening for PPD. The title is changed to “Risk of postpartum depression in Sri Lanka: A population-based study using a validated screening tool”.<br><br>Comment 2: Lines 17 & 40: The prevalence estimates provided here were 1996 figures... could there not have been much more recent ones? Also add the estimates from South Asia - as you compare it in the conclusion<br>Response: Good point. We have changed the reference on prevalence estimates in line 16 and line 104 to a more recent one by Hahn-Holbrook (2018). We also added prevalence ranges of PPD in South Asia in the introduction in line 73. However, we removed the sentence “this is lower than other countries in South Asia” in the conclusion as we have put more focus to screening for PPD in Sri Lanka in the revised version.<br><br>Comment 3: Lines 56 - 58: I am not clear about this point... what has this to do with PPD? I read over the cited article, did not come across a relationship between poverty and quality of parenting... please clarify...<br>Response: We have deleted this sentence.<br><br>Comment 4: Lines 92 - 93: Why the assumption based on 1996 figures? More so, when there was a 2017 study in the same population?<br>Response: At the time of planning and estimating our sample size we did not have the 2017 figures. As we knew that around 1000 births take place each calendar year in Galle, we estimated that in our sample 1000 mothers would give a reasonable precision and power. We calculated samples size both using the 1996 figures and using lower hypothetical figures with not related references. Basically our considerations tested more but only referred to the published old number. As we have now changed the 1996 reference in the introduction to a more recent one, we also updated the sample size estimation reference to be 18% Hahn-Holbrook (2018).<br><br>Comment 5: Lines 94 - 95: Why all mothers????? We the readers are not privy to information on deliveries in the study community... Also, the results section (line 159) has 975 persons... you have not clearly stated how was that arrived at... were those all the mothers interviewed? any mothers not included? response rate?<br>Response: Thank you for pointing this out. Basically all women who attended clinic after delivery are asked to fill the EPDS questionnaire screening them for postpartum depression. It is part of the routine public services at Galle, and we do not know whether any of them refused to fill the form, as we have only the filled forms. Exclusion criteria was incomplete EPDS records. We adjusted the methods section according to you recommendation, and added a line in the first paragraph in results section in line 177 to make this clearer.<br><br>Comment 6: Line 98: Will suggest this section be structured as e.g., data collection methods, tools, and process.<br>Response: Thank you for this great suggestion. We have now made the following subsections under methods “study design and study setting”, “participants”, “study tool”, “data collection”, “outcome measures and exposure variables”, “data analysis” and “ethical issues”.<br><br>Comment 7: Lines 106 - 107: check this.... specificity????<br>Response: Thank you for notifying this mistake. It has now been corrected in line 117-118.<br><br>Comment 8: Lines 109 - 115: Is the information presented here related to data collection?<br>Response: No, the information presented was related to the current practice on screening for PPD in Sri Lanka. Thank you for notifying this. We have now divided the methods section into more subheadings to separate our data collection from the study

Powered by Editorial Manager® and ProduXion Manager® from Aries Systems Corporation

tool according to your recommendation.

Comment 9: Line 114: Is the ICD used for classification or diagnosis?
Response: Yes, we have now changed the line 127 to be more precise.

Comment 10: Line 126: Label this as a sub-section e.g., variables considered.
Response: Thank you for this recommendation, we agree to add a subsection before this paragraph and have adjusted it accordingly.

Comment 11: Lines 159 - 162: Please make reference to table 2.
Response: Table 2 is now referred to in the text line 178.

Comment 12: Lines 193 - 195: Revise the title to be much more concise.
Response: We have shortened the title of table 2.

Comment 13: Line 201: Mention "EPDS Question 10" in the "Methods section" as part of the data collection tools.
Response: Thank you for this recommendation. We added this information under methods section in line 118.

Comment 14: Lines 214 - 216: Were these characteristics also in the 2017 study? If so, state that clearly... if not, then provide the appropriate citation.
Response: Thank you, this needed clarification. The factors in our study that showed the strongest association with PPD was former history of mental illness and death of the newborn, both not mentioned in the 2017 study. Hypertension was associated with PPD and not studied separately in the former study. High maternal age was associated with PPD in the former study, and this is mentioned in maternal age section in discussion.

Comment 15: Finally, please follow the reporting guidelines for Cross-sectional studies as provided in the link to guide your paper: https://www.equator-network.org/reporting-guidelines/strobe/. Also review the PLOS ONE guidelines on publications: https://journals.plos.org/plosone/s/criteria-for-publication
Response: Thank you for providing us with this information. We will adjust the paper according to the guidelines provided.

REVIEWER

Reviewer #1: The study addresses a key issue however there are some modifications required.

Background

Comment 16: The authors provide some useful information about postpartum depression in the background. Considering that a very similar study had already been conducted in the catchment area however, I think the authors could have provided more information on PPD in Sri Lanka and Galle. In addition, information on the validated tools used in measuring PPD in Sri Lanka as stated in the previous study are also relevant to this study. They could have been stated and appropriately referenced in this section as well.
For example, the fact that the EPDS have been translated into Sinhalese and validated in Sri Lanka is important information that should be made known in the background paper. Additionally, PPD in Sri Lanka is reported to have decreased since 2011 and the background of this paper should have captured this information.

Response: Thank you for these great suggestions. We agree that the findings from 2011 should have been included in the introduction and we have adjusted it accordingly. In the introduction we also added new information about use of cut-off scores when screening for PPD, as well as information the validated cut-off for screening in Sri Lanka and current practice.

Comment 17: Justification of study: Lines 71-74
The study was justified on the grounds that the similar study conducted in the same area in 2017 showed a PPD lower than that of another study conducted in Sri Lanka in

Powered by Editorial Manager® and ProduXion Manager® from Aries Systems Corporation

2004. However, a lot could have happened between 2004 to 2017 when the new study was conducted. Various reasons could have contributed to the difference and a proper review of the programs and interventions put in place between 2004 to 2017 could have provided more information as to why this difference was observed. Additionally, the 2017 paper indicated that there had been a general reduction in PPD from 2011, so the reduction should not really come as a surprise anyway. In my view therefore, there is not a strong justification for this study and it does not really offer anything new as it stands.

Response: We thank the reviewer for the very interesting comment. We would like to mention that the study from 2017 was published after we had collected our data in 2019 and the results were not available at the time when we made the research protocol. From your input we now realize that we should have been clearer on this issue, and not use the paper from 2017 as an argument for conducting a similar study over again as this study was not available at the time we gathered data. Nevertheless, we believe that our study provide new information about PPD in Sri Lanka as our study is the first study to use the validated cut-off 9 or more for screening for PPD in Sri Lanka. Previous studies on PPD in Sri Lanka have used the cut-off 10 or more for reasons we are not aware of. However, the validated cut-off for screening is 9 or more and we believe that our study provides more credibility to the findings on prevalence on PPD in Sri Lanka. Furthermore, our study focuses on individual risk factors including diseases during pregnancy as well as former history of mental illness, of which the 2017 study did not provide information. For future studies on postpartum and antenatal depression in Sri Lanka it is important to use the validated cut-off for screening for postnatal depression.

Comment 18: Recommendation: A comparative analysis or trend analysis between the previous studies from 2011 to 2017 will give a better justification for the study and provide a clearer picture of PPD in Sri Lanka. Therefore, if the data is available, I recommend that the authors provide some more information on PPD between 2011 to 2017. The authors can also make a stronger justification for the study using the timing of their administration of their EPDs if it differs significantly from other studies in Sri Lanka

Response: Thank you for good suggestions. In our study, the mothers are screened once at clinics for PPD between 4-6 weeks following delivery, not at two different timepoints. The 2017 study screened once for postpartum depression at 10 days following delivery in Dankotuwa district and once in Galle district 4 weeks following delivery. As two different times for screening was not completed within the same population, we believe that there are several sociodemographic confounding factors that could have contributed to these results. Furthermore, when testing after 10 days the findings of PPD prevalence may be confounded by the common “postpartum blues” which is limited to 2 weeks following delivery. We think by using 4 weeks instead of the first 10 days postpartum we give a better picture of the real depressive postpartum conditions that appear.

Methods

Comment 19: The collection of data using the EPD form is not really clear. Was the PPD information collected as part of the routine data collection or the team specifically went out to the field and collected this data using midwives?
This distinction should be made clearer in the write up. It is clear that the team made use of routine records for the other variables but as things stand now one gets the impression that the PPD information was collected in the same way. I am not sure that is the case however if the previous 2017 study is anything to go by
Response: Thank you for pointing this out. We agree and have adjusted the text to be clearer.

Comment 20: Additionally, if this is not the case then, I am also wondering why informed consent (verbal at least) was not required? Was this study simply a secondary analysis of routine data?
Response: The EPDS and pregnancy records are routinely administered in all mothers in Sri Lanka, meant for screening and potentially referral for further assessment and management. Therefore, as you have stated, the study is a secondary analysis of

	routine data. However, we understand that we were not clear on this and have adjusted the manuscript accordingly.<br><br>Comment 21: How often is screening done for PPD at the facility?<br>Response: The screening is completed once in Galle, Sri Lanka, and it is administered between 4-6 following delivery. This study digitalized the routine copies of the forms to analyze the data that is stored in the records of the clinic clients.<br><br>Comment 22: Was the EPD form only employed because of this current study? If the screening form and PPD data is readily available, then the team could also do a trend analysis or provide some more information on PPD in the catchment area prior to the study.<br>Response: Thank you for good suggestions. The EPDS is a part of a routine screening service. However, we are not in the possession of PPD data from years before, and accessing this data is not possible. Therefore, a trend analysis is unfortunately not possible to conduct.<br><br>Comment 23: Lines 104-109: The study made use of the Tamil and Sinhala versions of the EPDS. Additionally, the study also talks about illiterate participants. Who are the illiterate participants? Are illiterate participants in this case participants who could not read Tamil and Sinhala?<br>Response: Thank you for pointing this out. Agree, we have accordingly revised line 121-122 to emphasize who the illiterate participants are.<br><br>Results<br><br>Comment 24: The results are well presented. Some of the confidence intervals are really wide though and the authors should take a second look at their analyses and the assumptions made for their models. Example New born death aOR 28.9 (4.5-185.1). The authors did not present any findings from their chi square analysis.<br>Response: Thank you. We agree with this comment. As you know when the cases are few the confidence intervals of the estimates are wide, which is what we see with the mothers who lost their child. We prefer to give estimates and statistical significance shown as 95% confidence limits instead of p-values; however, if the editor insists, we can easily provide p-values in addition to the 95% CI.<br><br>Discussion<br><br>Comment 25: Line 209: According to the authors they dealt with women who had just given birth. However, in earlier chapters, they spoke about women who had given birth 4 to 6 weeks ago. The authors should be consistent as time is important in measurement of PPD. The study in 2017 for example had two time points (10 days and 4 weeks). Recommendation: In the 2017 study, it was recommended that further studies on the effect of time since delivery on PPD should be looked at. Since the form is administered routinely, the authors can look at the different time periods that the forms are administered to make a stronger case about PPD in Sri Lanka.<br>Response: We agree and we have adjusted the text to be clearer in line 230. Regarding effect of time since delivery, please see the response to comment 18 which explains why a study on different times for screening is not possible to conduct with the data we have available.<br><br>Conclusion<br><br>Comment 26: The conclusion here is much better than that the one in the abstract. The authors could look at revising the one in the abstract<br>Response: Thank you. We have adjusted the conclusion in the abstract accordingly.
**Additional Information:**	
**Question**	**Response**
**Financial Disclosure**<br><br>Enter a financial disclosure statement that describes the sources of funding for the	The authors received no specific funding for this work.

Powered by Editorial Manager® and ProduXion Manager® from Aries Systems Corporation

work included in this submission. Review the submission guidelines for detailed requirements. View published research articles from *PLOS ONE* for specific examples.<br><br>This statement is required for submission and **will appear in the published article** if the submission is accepted. Please make sure it is accurate.<br><br>**Unfunded studies**<br>Enter: *The author(s) received no specific funding for this work.*<br><br>**Funded studies**<br>Enter a statement with the following details:<br>• Initials of the authors who received each award<br>• Grant numbers awarded to each author<br>• The full name of each funder<br>• URL of each funder website<br>• Did the sponsors or funders play any role in the study design, data collection and analysis, decision to publish, or preparation of the manuscript?<br>• **NO** - Include this sentence at the end of your statement: *The funders had no role in study design, data collection and analysis, decision to publish, or preparation of the manuscript.*<br>• **YES** - Specify the role(s) played.<br><br>* typeset	
**Competing Interests**<br><br>Use the instructions below to enter a competing interest statement for this submission. On behalf of all authors, disclose any competing interests that could be perceived to bias this work—acknowledging all financial support and any other relevant financial or non-financial competing interests.<br><br>This statement is required for submission and **will appear in the published article** if the submission is accepted. Please make sure it is accurate and that any funding sources listed in your Funding Information	The authors have declared that no competing interests exist.

Powered by Editorial Manager® and ProduXion Manager® from Aries Systems Corporation

later in the submission form are also declared in your Financial Disclosure statement.<br><br>View published research articles from *PLOS ONE* for specific examples.<br><br>**NO authors have competing interests**<br><br>Enter: *The authors have declared that no competing interests exist.*<br><br>**Authors with competing interests**<br><br>Enter competing interest details beginning with this statement:<br><br>*I have read the journal's policy and the authors of this manuscript have the following competing interests: [insert competing interests here]*<br><br>* typeset	
**Ethics Statement**<br><br>Enter an ethics statement for this submission. This statement is required if the study involved:<br><br>• Human participants<br>• Human specimens or tissue<br>• Vertebrate animals or cephalopods<br>• Vertebrate embryos or tissues<br>• Field research<br><br>Write "N/A" if the submission does not require an ethics statement.<br><br>General guidance is provided below. Consult the submission guidelines for detailed instructions. **Make sure that all information entered here is included in the Methods section of the manuscript.**	Ethical clearance for the present study was obtained from Ethics Review Committee, Faculty of Medicine, University of Ruhuna (ID: 2019/P/055(09.10.2019)) and from REK, Regionale komiteer for medisinsk og helsefaglig forskningsetikk (ID: 77044). Informed consent was not required as this short questionnaire was used as routine screening service and introduced into antenatal clinics several years before. The data archive had no names or identifiers.

Powered by Editorial Manager® and ProduXion Manager® from Aries Systems Corporation

**Format for specific study types**<br><br>**Human Subject Research (involving human participants and/or tissue)**<br>• Give the name of the institutional review board or ethics committee that approved the study<br>• Include the approval number and/or a statement indicating approval of this research<br>• Indicate the form of consent obtained (written/oral) or the reason that consent was not obtained (e.g. the data were analyzed anonymously)<br><br>**Animal Research (involving vertebrate animals, embryos or tissues)**<br>• Provide the name of the Institutional Animal Care and Use Committee (IACUC) or other relevant ethics board that reviewed the study protocol, and indicate whether they approved this research or granted a formal waiver of ethical approval<br>• Include an approval number if one was obtained<br>• If the study involved *non-human primates*, add *additional details* about animal welfare and steps taken to ameliorate suffering<br>• If anesthesia, euthanasia, or any kind of animal sacrifice is part of the study, include briefly which substances and/or methods were applied<br><br>**Field Research**<br><br>Include the following details if this study involves the collection of plant, animal, or other materials from a natural setting:<br>• Field permit number<br>• Name of the institution or relevant body that granted permission	
**Data Availability**<br><br>Authors are required to make all data underlying the findings described fully available, without restriction, and from the time of publication. PLOS allows rare exceptions to address legal and ethical concerns. See the PLOS Data Policy and FAQ for detailed information.	Yes - all data are fully available without restriction

A Data Availability Statement describing where the data can be found is required at submission. Your answers to this question constitute the Data Availability Statement and **will be published in the article**, if accepted.<br><br>**Important:** Stating 'data available on request from the author' is not sufficient. If your data are only available upon request, select 'No' for the first question and explain your exceptional situation in the text box.<br><br>Do the authors confirm that all data underlying the findings described in their manuscript are fully available without restriction?	
**Describe where the data may be found in full sentences. If you are copying our sample text, replace any instances of XXX with the appropriate details.**<br><br>• If the data are **held or will be held in a public repository**, include URLs, accession numbers or DOIs. If this information will only be available after acceptance, indicate this by ticking the box below. For example: *All XXX files are available from the XXX database (accession number(s) XXX, XXX.*).<br>• If the data are all contained **within the manuscript and/or Supporting Information files**, enter the following: *All relevant data are within the manuscript and its Supporting Information files.*<br>• If neither of these applies but you are able to provide **details of access elsewhere**, with or without limitations, please do so. For example:<br><br>*Data cannot be shared publicly because of [XXX]. Data are available from the XXX Institutional Data Access / Ethics Committee (contact via XXX) for researchers who meet the criteria for access to confidential data.*<br><br>*The data underlying the results presented in the study are available from (include the name of the third party*	All relevant data are within the manuscript and its Supporting Information files.

and contact information or URL).<br>• This text is appropriate if the data are owned by a third party and authors do not have permission to share the data.<br><br>* typeset	
Additional data availability information:	

October 06, 2021

Dear Editor,

We are grateful to the good reviewer for great suggestions, most of which we agree to and have consequently modified numerous sections. We attach a document with all comments from you and reviewers, and inserted our specific responses to each issue that was raised. We also attach a new clean version without track changes. The data set has been uploaded in Supporting Information files and there are no restrictions on sharing the data set.

We think the document is better now, and send this for you to assess whether it can be published in the improved version.

On behalf of all the authors,

Sincerely,
Therese Røysted Solås

Manuscript Click here to access/download;Manuscript;Manuscript.docx

Risk of postpartum depression in Sri Lanka: A population-based study using a validated screening tool

Therese Røysted Solås [1*], Sven Gudmund Hinderaker [1], Lasantha Ubesekara [2], Vijitha De Silva [3]

[1]University of Bergen, Bergen, Norway

[2]Ministry of Health, Southern Province, Sri Lanka

[3]University of Ruhuna, Galle, Sri Lanka

* Corresponding author

E-mail: therese.solas@student.uib.no

Abstract

Background: Postpartum depression is an important public health concern. The prevalence of postpartum depression is estimated to be 18% worldwide. The purpose of this study was to estimate the prevalence of postpartum depression in Sri Lanka and to investigate its associated risk factors.

Methods: This was a cross-sectional study conducted among 975 mothers in Galle district, Sri Lanka. The prevalence of mothers at risk of postpartum depression was assessed using the Edinburgh Postpartum Depression scale (EPDS) which has been validated for screening for postpartum depression in Sri Lanka with a cut-off score 9 or more. Data from routine records on pregnancy, delivery, postnatal care was collected to investigate possible predictors of postpartum depression. Crosstabulation and multivariable logistic regression was performed to identify risk factors for postpartum depression, defined as EPDS score 9 or more. Prevalence estimates using different cut-offs for postpartum depression was compared to assess the impact of using unvalidated cut-offs for screening.

Results: The prevalence of mothers with EPDS score 9 or more was found to be 9.4% (95%CI: 7.8-11.4); EPDS score 10 or more was 5.6% (95%CI: 4.4-7.3). Postpartum depression (EPDS 9 or more) was associated with the following risk factors: Former history of mental illness (aOR 32.9, 95%CI: 7.9-136.2), high maternal age 30-39 (aOR 2.2, 95%CI: 1.3-3.8), BMI 25.0-29.9 (aOR 2.6, 95%CI: 1.5-4.5), hypertension (aOR 3.6, 95%CI: 1.2-10.9) and newborn death (aOR 28.9, 95%CI: 4.5-185.1). One in five women reported thoughts of self-harm.

Conclusion: A significant proportion of Sri Lankan mothers experience symptoms of postpartum depression, highest risk among mothers who reported former history of mental illness and newborn death. Using a higher cut-off for screening had a substantial impact on the prevalence rate estimates. Mothers at increased risk should be identified in antenatal care and is an important target of referral.

Introduction

Depression after giving birth is considered a serious public health problem worldwide, and the overall prevalence worldwide is estimated to be 18% [1]. Studies suggest that postpartum depression (PPD) not only affects the wellbeing of postpartum women, but also the mother-infant interaction [2]. There is a compelling body of evidence that PPD may influence the social, cognitive and emotional development of the child and infant growth [2].

PPD and its associated risk factors are well studied in high income countries (HICs) but data from low- and middle-income countries (LMICs) is scarce [3]. However, research suggest there is a higher prevalence of PPD in LMICs compared to in HICs [3], which aligns well with low socioeconomic status as a known risk factor [2]. It is widely recognized that PPD is not attributable to one single cause, but rather influenced by complex interactions between environmental, psychosocial and biological risk factors [2]. Several risk factors associated with PPD have been investigated. PPD in mothers is more often observed in those with a former history of mental illness [4]. Associations with maternal age have shown conflicting results [5]. Furthermore, previous research suggests that thoughts of self-harm in pregnancy is not uncommon [6], as in many populations.

Screening for and managing PPD is included in *The Global Strategy for women's, children's and adolescent's health (2016-2030*) by the World Health Organization [7]. Several tools to screen or diagnose PPD have been used, and this is reflected in the diversity in reported prevalence in different countries [5]. The Edinburgh Postnatal Depression Scale (EPDS) is a 10-item questionnaire and the most widely used tool globally to screen for mothers at risk of PPD [2]. Each answer is scored 0-3 and a higher sum of scores indicates more symptoms of depression. Originally, the EPDS was developed for screening for PPD in English-speaking women and a cut-off 10 or more for "possible depression" and a cut-off 13 or more for "probable depression" was suggested [8]. Since then, the EPDS has been translated to and validated in many languages

using different cut-offs for screening in different countries. Optimal psychometric properties are important for assessing prevalence rates and the overall burden of PPD in countries [9]. In 2006, Matthey et al. put focus on the increasing use of unvalidated EPDS cut-off scores in studies on PPD [9]. Furthermore, he stated that a difference of just 1 point in cut-off score can have a substantial impact on estimates of PPD rates [9].

In South-Asia, the prevalence of PPD have shown to range from 5-49% [1]. Sri Lanka is a lower middle-income country and has been considered a role model for maternal health promotion and for reducing maternal deaths. In Sri Lanka the Sinhala version of the EPDS was introduced and included as a part of postnatal care in 2012 [10] and it has been validated for screening for PPD in mothers with a cut-off 9 or more for depression [11]. In 2011, a cross sectional study representing eight provinces in Sri Lanka estimated the prevalence of PPD to be 27.1% using a cut-off 10 or more [12]. A recent study from 2017 in Galle, Sri Lanka, found a that 7.8% of mothers had an EPDS score 10 or more [13]. To this date there are no published studies available on the prevalence of mothers at risk of PPD in Sri Lanka using the validated cut-off for screening EPDS score 9 or more. Using a higher cut-off may lead to an underestimation of the burden of PPD in Sri Lanka. Therefore, in our study we aimed to: 1) repeat this study on EPDS score in postpartum women at antenatal clinics in Galle using an EPDS cut-off 9 or more 2) evaluate the difference on prevalence estimates using a cut-off 10 or more 3) investigate risk factors associated with the validated cut-off for screening of PPD of EPDS score 9 or more among pregnant women.

Methods

Study design and study setting

A cross sectional study was conducted among mothers who had delivered from 1st January 2019 to 31st December 2019 in Bope-Poddala, Medical Offices of Health (MOH) Division, Galle district,

Sri Lanka. With a population of over 71 000, Bope-Poddala is a semi urban MOH division in Galle district. All three main population groups of Sri Lanka live there: Sinhalese, Tamils and Muslims. Here preventive care services are provided through 20 divisions of the MOH. Antenatal and postnatal care is provided to the mothers through five maternal and child health field clinics. In Sri Lanka the antenatal care attendance is very high, and nearly 100% of deliveries occur in health facilities. Maternal mental health services are provided during regular visits in the clinics by a medical officer of mental health.

Participants

In Bope-Poddala, around one thousand births take place each calendar year. The sample size for the postpartum mothers to be included in the study was estimated using OpenEpi.com calculator [14]. Assuming that the prevalence of PPD in Galle would be around 18% [1] and aiming at estimating the proportion with a precision of +/- 2.5%, the required sample size for our study was estimated to be n=907. Therefore, all the postpartum mothers who delivered from 1st January 2019 to 31st December 2019 were included in the study. Exclusion criteria was incomplete EPDS records.

Study tool

In Sri Lanka, all mothers are routinely screened once at the clinics for PPD 4 to 6 weeks after delivery using Sinhala or Tamil version of Edinburgh Postpartum Depression Scale (EPDS). An EPDS score 9 or more for depression has been validated for screening for PPD with the Sinhala version of the EPDS in Sri Lanka, with a sensitivity and specificity of 89.9% and 78.9%, respectively [11]. The Tamil version of the EPDS is used for screening in the Tamil population. The Tamil version of the EPDS has been validated for screening in Tamil Nadu, India using the same values for depression, EPDS 9 or more, with a sensitivity of 94.1% and a specificity of 90.2% [15]. The EPDS question 10 is an item on thoughts of self-harm.

In current practice, the EPDS is filled on paper forms by the mothers at their postnatal visit at the clinic and then collected and kept by the midwives. If the mother cannot read Tamil or Sinhala the EPDS is completed by the midwife through interview with the mother. Mothers with an EPDS score 9 or more are referred to a medical officer of health at the clinics who then refer the mother to their designated medical officer of mental health (MOMH). MOMH manage some of the cases, and if necessary, refers the mother to psychiatrist care at the nearest hospital. In severe cases the medical officer of health refers directly to a consultant psychiatrist. MOMHs and psychiatrist diagnose mothers with PPD using the ICD-10 classification for diagnosis, usually through clinical interview with the mother.

Data collection

All pregnant women have EPDS records and routine records on pregnancy, delivery, postnatal care in the B part of the pregnancy record which is kept in the clinics. For our study, determinants were collected as quantitative clinical data from these records into a data extraction sheet by a trained research assistant. The data extraction sheet included sociodemographic, obstetric and baby related variables. The collected data was translated to English and double-entered into the electronic database EpiData 3.1. After ensuring that data from various sources were linked to the same person, the names and identifiers were removed. In Sri Lanka, pregnancy outside marriage is very rare and thus there is no place for marital status in the pregnancy record. Local midwives had a way of knowing, but could not identify anyone unmarried in the study participants. However, this is associated with uncertainty.

Outcome measures and exposure variables

The main outcome measurement was rate of mothers with EPDS score 9 or more. Exposure variables were: maternal age (under 20, 20-29, 30-39 and 40 and more); educational level

(completed secondary school ordinary level or below, completed above secondary school ordinary level); occupation (housewife or non-housewife); socioeconomic status (calculated according to the occupation of the mother and the husband, and categorized into SE 1-2 and SE 3-5); number of living children (0, 1, 2-3 and >4); former history of mental illness (yes/no); maternal Body Mass Index (BMI) (less than 18.5, 18.5-24.9, 25.0-29.9 and 30 and more); hypertension during pregnancy (yes/no); diabetes during pregnancy (yes/no); history of subfertility (yes/no); death of newborn defined as death of liveborn within 28 days (yes/no); gender of the baby (female or male); congenital abnormalities (yes/no); birthweight (less than 2500g, 2500g or more); establishment of breastfeeding (yes/no); other complications from delivery notes (yes/no).

Data analysis

The database was exported into IBM SPSS 26 version for statistical analysis. The prevalence of mothers at risk of PPD was calculated as percentages of mothers with an EPDS score 9 or more. Differences in categorical variables according to the prevalence of EPDS score 9 or more were analyzed with Pearson's Chi-square test (corrected by Fisher exact test for small samples) and level of significance was set at $p<0.05$. One-way ANOVA was performed to compare the mean EPDS score for each independent variable. Possible predictors of PPD were examined in univariate and multivariable models. In the final model we adjusted with maternal age, BMI, former history of mental illness, hypertension, history of subfertility and newborn death. Odds ratios (OR) and adjusted Odds ratios (aOR) with 95% confidence intervals (95%CI) were calculated. The prevalence of mothers reporting thoughts of self-harm was calculated as percentages of mothers reporting "never", "hardly ever", "sometimes" and "many times" on the EPDS item 10.

Ethical issues

Ethical clearance for the present study was obtained by Ethics Review Committee, Faculty of Medicine, University of Ruhuna and by REK, Regionale komiteer for medisinsk og helsefaglig forskningsetikk. Informed consent was not required as the EPDS was used as routine screening service and introduced into antenatal clinics several years before. The data archive had no names or identifiers.

Results

Included in the study were 975 pregnant women. All mothers had complete EPDS records and no mothers were excluded. Table 2 shows the characteristics of the mothers. Among the respondents the mean age was 29 years, ranging from 16 to 45 years. The majority of the mothers (70.5%) were housewives, and half of the mothers (48.1%) had completed education above ordinary level examination secondary school. Former history of mental illness was identified in 12 women (1.2%).

Table 1 shows the EPDS scores among the 975 women studied; 9.4% (n=92) had an EPDS score 9 or more; and 5.6% (n=55) had a EPDS score 10 or more. An EPDS score 12 or more was seen in 2.1% (n=20) of the participants.

Table 1. Frequency distribution of the EPDS scores with sensitivity analysis among mothers in Galle, 2019

EPDS	Frequency (n)	Percent (%)	95%CI
Total	975	100	-
EPDS score 9 or more	92	9.4	7.8-11.4
EPDS score 10 or more	55	5.6	4.4-7.3
EPDS score 11 or more	29	3.0	2.1-4.2
EPDS score 12 or more	20	2.1	1.3-3.1

Association of postpartum depression with risk factors

Table 2 shows mean EPDS score by exposure variables and corresponding logistic regression analysis of these determinants of PPD, defined as EPDS score 9 or more. Compared to mothers aged 20-29, those aged 30-39 had twice the risk of PPD (aOR 2.2, 95%CI: 1.3-3.8). Mothers who had experienced newborn death had a higher risk of PPD (aOR 28.9, 95%CI: 4.5-185.1), and mothers who reported a former history of mental illness had a highly increased risk of PPD (aOR 32.9, 95%CI: 7.9-136.2). Mothers with a BMI <18.5 (aOR 3.0, 95%CI: 1.4-6.3) and BMI 25.0-29.9 (aOR 2.6, 95%CI: 1.5-4.5) had a higher risk of PPD than those with normal BMI. Those who had hypertension were 3.6 times more likely (aOR 3.6, 95%CI: 1.2-10.9) to develop PPD as compared to mothers without hypertension.

No significant difference was found between the occurrence of PPD and obstetric factors. Baby gender and late breast-feeding initiation was not significantly associated with the development of PPD. PPD defined as EPDS score 10 or more showed similar associations with the same variables (not included in the table).

Table 2. Determinants of postpartum depression (EPDS score 9 or more) among mothers in Galle, 2019

Variables	Total	EPDS 9 or more	Mean EPDS (95% CI)	OR (95% CI)	aOR (95% CI)
Maternal age					
≤19	52	2	4.6 (4.0-5.2)	0.6 (0.1-2.6)	0.8 (0.2-3.7)
20-29	459	29	3.8 (3.6-4.1)	1	1
30-39	447	59	**4.6 (4.3-4.9)**	**2.3 (1.4-3.6)**	**2.2 (1.3-3.8)**
≥40	17	2	4.2 (2.6-5.9)	2.0 (0.4-9.0)	1.0 (0.1-8.6)
Education					
≤Ordinary level	505	46	4.4 (4.1-4.6)	1	1
>Ordinary level	469	46	4.1 (3.8-4.4)	1.1 (0.7-1.7)	1.1 (0.6-1.7)
Missing	1				
Occupation					
Housewife	687	68	4.3 (4.1-4.6)	1	1
Non-housewife	288	24	4.0 (3.7-4.3)	0.8 (0.5-1.3)	0.7 (0.4-1.2)
Socioeconomic class					
1-2	194	13	3.6 (3.3-4.0)	0.6 (0.3-1.2)	0.6 (0.3-1.2)
3-5	781	79	4.4 (4.2-4.6)	1	1
Number of living children					
0	0	0	--	-	-
1	428	34	4.1 (3.8-4.4)	1	1
2-3	489	52	4.3 (4.0-4.5)	1.4 (0.9-2.2)	0.8 (0.4-1.3)
>4	57	6	4.5 (3.9-5.2)	1.4 (0.5-3.4)	0.5 (0.2-1.4)
Missing	1				
History of mental illness					

Yes	12	9	**11.0 (8.1-13.9)**	**31.8 (8.4-119.8)**	**32.9 (7.9-136.2)**
No	963	83	4.1 (4.0-4.3)	1	1
BMI					
≤18.4	119	13	**4.4 (3.8-5.0)**	**2.0 (1.0-4.0)**	**3.0 (1.4-6.3)**
18.5-24.9	462	27	4.0 (3.7-4.2)	1	1
25.0-29.9	290	43	**4.6 (4.2-4.9)**	**2.8 (1.7-4.7)**	**2.6 (1.5-4.5)**
≥30	57	3	4.1 (3.4-4.9)	0.9 (0.3-3.1)	0.8 (0.2-2.8)
Missing	47				
Hypertension					
**Yes**	17	6	**6.3 (4.4-8.1)**	**5.5 (2.0-15.3)**	**3.6 (1.2-10.9)**
No	958	86	4.2 (4.0-4.4)	1	1
Diabetes					
Yes	54	8	5.0 (4.0-5.9)	1.7 (0.8-3.8)	0.7 (0.2-1.9)
No	921	84	4.2 (4.0-4.4)	1	1
History of subfertility					
Yes	39	7	5.3 (4.0-6.5)	2.2 (0.9-5.1)	1.0 (0.4-2.8)
No	936	85	4.2 (4.0-4.4)	1	1
Newborn death					
Yes	6	4	**9.0 (4.6-13.4)**	**20.0 (3.6-110.9)**	**28.9 (4.5-185.1)**
No	969	88	4.2 (4.0-4.4)	1	1
Baby gender					
Male	487	41	4.2 (3.9-4.4)	0.8 (0.5-1.2)	0.7 (0.4-1.2)
Female	487	51	4.3 (4.0-4.6)	1	1
Missing	1				

Congenital abnormalities					
Yes	8	2	6.0 (2.1-9.9)	3.2 (0.6-16.3)	1.3 (0.1-11.8)
No	966	90	4.2 (4.0-4.4)	1	1
Missing	1				
Birthweight					
<2500	89	6	4.3 (3.7-5.0)	0.7 (0.3-1.6)	0.5 (0.2-1.3)
≥2500	886	86	4.2 (4.0-4.4)	1	1
Breast feeding					
Yes	429	35	4.2 (4.0-4.5)	0.8 (0.5-1.2)	0.6 (0.4-1.1)
No	546	57	4.2 (4.0-4.5)	1	1
Delivery complication					
Yes	40	4	4.6 (3.4-5.8)	1.1 (0.4-3.1)	0.5 (0.2-2.0)
No	934	88	4.2 (4.0-4.4)	1	1
Missing	1				
Baby birth complication					
Yes	23	2	5.0 (3.4-6.5)	0.9 (0.2-3.9)	0.1 (0.0-1.4)
No	936	90	4.2 (4.0-4.4)	1	1
Missing	16				
Malpresentation					
Yes	45	1	4.1 (3.3-5.0)	0.2 (0.0-1.5)	0.1 (0.0-1.3)
No	929	91	4.2 (4.0-4.4)	1	1
Missing	1				

** Analysed by ANOVA with mean EPDS score, and multivariable logistic regression with odds ratios.* *Regression analysis included these variables in the adjustment model: maternal age, BMI, former history of mental illness, hypertension, history of subfertility and newborn death. Other variables were adjusted with the same model.*

Thoughts of self-harm

Table 3 shows responses to EPDS Question 10 and the associated mean total EPDS. Nearly 1 out of 5 (n=188) study participants reported “the thought of harming myself has occurred to me”. Out of those 188 participants, 26.1% had an EPDS score 9 or more. We see that the more frequent such thoughts are reported, the more likely depression occurred.

Table 3. EPDS question 10: “The thought of harming myself has occurred to me”

Response	Frequency (n)	Percent (%)	Mean total EPDS (95%CI)
Total	*975*	*100*	-
Never	*787*	*80.7*	3.6 (3.4-3.8)
Hardly ever	*174*	*17.8*	6.6 (6.2-7.0)
Sometimes	*14*	*1.4*	8.3 (7.0-9.6)
Many times	*0*	*0*	-

Discussion

In this study in Sri Lanka among 975 women who had given birth between 4 to 6 weeks ago we found that 9.4% (95%CI: 7.8-11.4) had an EPDS score 9 or more, which means they are referred to mental health services for further management and diagnosis of PPD. The factor that showed the strongest association with PPD was former history of mental illness, and this was not investigated in the former publication. Several maternal characteristics and factors were associated with PPD: high maternal age, overweight, underweight, hypertension and newborn death.

Various studies on PPD in Sri Lanka have shown a wide variation in prevalence, perhaps partly due to differences in methods. In 2017 a similar study conducted in Galle, Sri Lanka reported

that 7.8% mothers had an EPDS score 10 or more [13], compared to 5.6% (95%CI: 4.4-7.3) in our material. Our findings support the previous finding of low risk of PPD in Galle, Sri Lanka. However, we see that when measuring the prevalence rates of PPD using a EPDS cut-off 9 or more compared to a cut-off 10 or more a difference of 3.8% in prevalence estimates occurred. This supports the statement by Matthey et. al that a difference of just 1 point in cut-off score can have a substantial impact on postpartum depression rates and highlights the importance of using the validated cut-off for screening in Sri Lanka [9]. On human development index, Galle ranked between mid and top on the health index and high on education index in Sri Lanka in 2013 [16] which may contribute to the low prevalence. Furthermore, the prevalence is lower than findings in other countries in South-Asia. A validation study from Nepal showed a prevalence of PPD at 17.1%. It defined depression as EPDS 13 or more and had a sensitivity of 92% and a specificity of 95.6% [17].. A meta-analysis from India estimated a pooled prevalence of PPD at 22% [18]. Compared to the neighboring countries, Sri Lanka have made more progress on maternal health and child mortality indicators, as well as other indicators such as GDP per capita and education [19].

In our study, mothers aged 30-39 had twice the risk of developing depressive symptoms compared to those aged 20-29. The same findings are reported in the paper from the same place in 2017 [13]. A higher percentage of EPDS score 9 or more in the age group 30-39 compared to other age groups was observed in both primiparous mothers and in multiparous mothers. Other sociodemographic risk factors such as educational level and being a housewife did not show significant associations with PPD.

In terms of maternal BMI, underweight women (BMI<18.5) had a three times risk of PPD compared to women of normal weight, which is consistent with findings in Nepal [20] and Sweden [21]. It is widely recognized that BMI is likely to be influenced by income [16] and adjusting for only socioeconomic class, the association between underweight and PPD was no

longer significant. In Sri Lanka, overweight (BMI 25.0-29.9) is far more prevalent than underweight and was in our study associated with more than twice the risk of developing PPD compared to mothers with normal bodyweight. One study suggest that overweight in Sri Lanka is associated with urban living and being in middle age [22].

We found that hypertension was associated with increased risk of PPD. In our study, the mothers with hypertension were more often of advanced age, which reflects the prevalence of hypertension in the general population [23]. Also, hypertensive mothers had more gestational diabetes, also increasing with age. The possible relationship between preeclampsia and PPD has previously been described in studies from South Korea [24] and Netherlands [25]. In pre-eclampsia, the blood level of serotonin is known to increase [26] and previous studies have hypothesized that this may be linked to a decreased level of serotonin in the brain [25]. A possible role of alterations in serotonin function has been noted in the pathophysiology of general depression [27]. A further complication may be that hypertension in pregnancy is commonly treated with methyldopa, of which a known side effect is depressive symptoms [28]. However, hypertension and preeclampsia could not be differentiated from the pregnancy record in our study.

Thoughts of self-harm was reported by almost 20%, of whom the vast majority responded "hardly ever". In a randomized controlled trial in the UK, it was found that the response "hardly ever" on EPDS item 10 in mothers was not concordant with suicidality as measured by a clinical interview [29]. A study published in 2019 which included 475 pregnant women in Anuradhapura, Sri Lanka reported rates on thoughts of self-harm at 5.9% including "hardly ever" [30], and in Goa, India, it was 14.3% [31],[6]. In Pittsburgh, USA, with a study population of 10 000 mothers, the observed rate on thoughts of self-harm 4 to 6 weeks postpartum was 3.2%, out of whom the majority had screen-positive findings on depression [32]. This is in contrast to our study, where only 1 out of 4 women with thoughts about self-harm had in addition an EPDS

score 9 or more; however, we noticed that the more frequent the mothers' thoughts were, the more signs of depression occurred, shown by increasing mean EPDS score. In Sri Lanka, mothers are usually not referred to a psychiatrist based on the response on EPDS item 10. However, research on self-harm behavior in Sri Lanka is limited and it remains to be shown whether self-harm ideation as measured by the EPDS item 10 can predict physical self-harm in Sri Lankan mothers.

In our study mothers who had experienced death of a newborn more often had EPDS score 9 or more, although grief reactions may be difficult to distinguish from PPD symptoms. We also found that former history of mental illness predicts PPD, which is consistent with the findings by O'Hara and Swain [4]. In our study, a diagnosis of mental illness before the pregnancy was included but not further defined. However, the number of mothers identified (n=12) may not represent the total number of mothers who ever had a mental illness; perhaps those with a more recent diagnosis were more likely to report on this item but we do not have this information. In Sri Lanka, stigmatizing beliefs on mental health have been reported to affect help-seeking behavior [33]. As in many Asian cultures, emotional restraint and self-control are perceived as desirable qualities in women, and to seek counselling for mental health issues may be considered unacceptable by family members [33]. Our findings suggest that attendants reporting a past mental illness may be considered at risk of PPD, and may benefit from referral.

Our study had several strengths. The adequate sample size provides some confidence to the precision in our results, and missing data was minimal. The transfer of information in paper forms into digital data was validated by double data entry. We think the study gives a good representation of the situation for pregnant women in the area, as almost all pregnant women there attend ANC. Furthermore, our study is the first study since the EPDS was included as a part of postnatal care in 2012 to use the validated cut-off for screening when estimating mothers at

risk of PPD in Sri Lanka.

The study also had some limitations. The cross-sectional study design can only analyze associations and not causation, but this is in line with our aims. Recall is generally associated with some uncertainty, especially about feelings in the past, but limiting to the past week may provide more credibility to the findings. Misinterpretation of the questions and social taboos may have led to response bias, but the local author did not see such obvious mistakes. The cut-off threshold for categorizing depression is a matter of discussion; we used a validated cut-off for screening for PPD in Sri Lanka, and we also tested the association with other cut-offs as well (Table 1) and found similar associations (not shown in table). The tool we used was tested and validated in both Sinhala and Tamil language. Nonetheless, great caution must be taken when comparing the results of EPDS screening for PPD in different countries as there is no international standard for how to translate, nor validate, the EPDS in local languages [34].

Conclusion

The prevalence of EPDS score 9 or more, indicating risk of PPD, in Galle, Sri Lanka was estimated to be 9.4% (95%CI: 7.8-11.4). A higher cut-off for screening had a substantial impact on the prevalence rate estimates and thus highlights the importance of using the validated cut-off EPDS score 9 or more for future studies on PPD in Sri Lanka. The major predisposing factors for postpartum depression were former history of mental illness, high maternal age, overweight, hypertension and newborn death. Furthermore, the number of mothers reporting thoughts of self-harm was high. Mothers at increased risk of PPD is an important target of referral to specialist.

Aknowledgements

The authors would like to thank all Public Health midwives of Bope-Poddala MOH division for their effort and cooperation in collecting data for this study, as well as our research assistant Ms. Prabodha Lakmali. We would like to thank Prof. Ganesh Acharya for his insightful suggestions and reading of the manuscript. This research was supported by University of Ruhuna, Sri Lanka and University of Bergen, Norway.

References

1. Hahn-Holbrook J, Cornwell-Hinrichs T, Anaya I. Economic and Health Predictors of National Postpartum Depression Prevalence: A Systematic Review, Meta-analysis, and Meta-Regression of 291 Studies from 56 Countries. Front Psychiatry. 2018;8. doi:10.3389/fpsyt.2017.00248
2. Stewart DE, Robertson E, Dennis C-L, Grace SL, Wallington T. Postpartum depression: Literature review of risk factors and interventions. Toronto: University Health Network Women's Health Program for Toronto Public Health. 2003.
3. Fisher J, Cabral de Mello M, Patel V, Rahman A, Tran T, Holton S, et al. Prevalence and determinants of common perinatal mental disorders in women in low- and lower-middle-income countries: a systematic review. Bull World Health Organ. 2012;90: 139–149. doi:10.2471/BLT.11.091850
4. O'Hara MW, Swain AM. Rates and risk of postpartum depression-a meta-analysis. Int Rev Psychiatry. 1996;8: 37–54. doi:10.3109/09540269609037816
5. Norhayati MN, Hazlina NH, Asrenee AR, Emelin WM. Magnitude and risk factors for postpartum symptoms: a literature review. J Affect Disord. 2015;175: 34–52. doi:10.1016/j.jad.2014.12.041
6. Lindahl V, Pearson JL, Colpe L. Prevalence of suicidality during pregnancy and the postpartum. Arch Womens Ment Health. 2005;8: 77–87. doi:10.1007/s00737-005-0080-1
7. Kuruvilla S, Bustreo F, Kuo T, Mishra CK, Taylor K, Fogstad H, et al. The global strategy for women's, children's and adolescents' health (2016-2030): A roadmap based on evidence and country experience. Bull World Health Organ. 2016;94: 398–400. doi:10.1016/b978-0-444-89007-8.50010-1
8. Cox J, Holden J, Sagovsky R. Detection of Postnatal Depression: Development of the 10-item Edinburgh Postnatal Depression Scale. BJPsych. 1987;150: 782-786. doi:10.1192/bjp.150.6.782.
9. Matthey S, Henshaw C, Elliott S, Barnett B. Variability in use of cut-off scores and formats

on the Edinburgh Postnatal Depression Scale - Implications for clinical and research practice. Arch Womens Ment Health. 2006;9: 309–315. doi:10.1007/s00737-006-0152-x

10. Agampodi SB, Agampodi TC. Antenatal Depression in Anuradhapura, Sri Lanka and the Factor Structure of the Sinhalese Version of Edinburgh Post Partum Depression Scale among Pregnant Women. PLoS One. 2013;8. doi:10.1371/journal.pone.0069708

11. Rowel D, Jayawardena P, Fernando N. Validation of the Sinhala translation of Edinburgh Postnatal Depression Scale. Ceylon Med J. 2008;53: 10–13. doi:10.4038/cmj.v53i1.219

12. Agampodi TC, Agampodi SB, Wickramasinghe WA, Adhikari AM, Chathurani HK. Post partum depression - a problem that needs urgent attention. Ceylon Med J. 2011;56: 183–184. doi:10.4038/cmj.v56i4.3907

13. Fan Q, Long Q, De Silva V, Gunarathna N, Jayathilaka U, Dabrera T, et al. Prevalence and risk factors for postpartum depression in Sri Lanka: A population-based study. Asian J Psychiatr. 2020;47: 101855. doi:10.1016/j.ajp.2019.101855

14. Dean AG, Sullivan KM, Soe MM. OpenEpi: Open Source Epidemiologic Statistics for Public Health, Version [cited 17 May 2021]. Available from: www.OpenEpi.com, updated 6 April 2013.

15. Benjamin D, Chandramohan A, Annie IK, Prasad J, Jacob KS. Validation of the Tamil version of Edinburgh post-partum depression scale. J Obstet Gynecol India. 2005;55: 241–243.

16. United Nations Development Programme. Sri Lanka Human Development report 2012. UNDP Sri Lanka, 2012.

17. Bhusal BR, Bhandari N, Chapagai M, Gavidia T. Validating the Edinburgh Postnatal Depression Scale as a screening tool for postpartum depression in Kathmandu, Nepal. Int J Ment Health Syst. 2016;10: 71. doi:10.1186/s13033-016-0102-6

18. Upadhyay RP, Chowdhury R, Salehi A, Sarkar K, Singh SK, Sinha B, et al. Postpartum depression in India: a systematic review and meta-analysis. Bull World Health Organ. 2017;95: 706-717B. doi:http://dx.doi.org/10.2471/BLT.17.192237

19. The World Bank. World Development Indicators, The World Bank Group, 2020 [cited 12 May 2021]. Available from: https://www.worldbank.org/en/home.

20. Ho-Yen SD, Bondevik GT, Eberhard-Gran M, Bjorvatn B. Factors associated with depressive symptoms among postnatal women in Nepal. Acta Obstet Gynecol Scand. 2007;86: 291–297. doi:10.1080/00016340601110812

21. Silverman ME, Smith L, Lichtenstein P, Reichenberg A, Sandin S. The association between body mass index and postpartum depression: a population-based study. J Affect Disord. 2018;240: 193–198. doi:10.1016/j.jad.2018.07.063

22. Katulanda P, Jayawardena MAR, Sheriff MHR, Constantine GR, Matthews DR. Prevalence of overweight and obesity in Sri Lankan adults. Obes Rev. 2010;11: 751–756. doi:10.1111/j.1467-789X.2010.00746.x

23. Katulanda P, Ranasinghe P, Jayawardena R, Constantine GR, Rezvi Sheriff MH, Matthews DR. The prevalence, predictors and associations of hypertension in Sri Lanka: a cross-sectional population based national survey. Clin Exp Hypertens. 2014;36: 484–491. doi:10.3109/10641963.2013.863321

24. Youn H, Lee S, Han SW, Kim LY, Lee TS, Oh MJ, et al. Obstetric risk factors for depression during the postpartum period in South Korea: a nationwide study. J Psychosom Res. 2017;102: 15–20. doi:10.1016/j.jpsychores.2017.09.003

25. Blom EA, Jansen PW, Verhulst FC, Hofman A, Raat H, Jaddoe VWV, et al. Perinatal complications increase the risk of postpartum depression. The Generation R Study. BJOG. 2010;117: 1390–1398. doi:10.1111/j.1471-0528.2010.02660.x

26. Bolte AC, van Geijn HP, Dekker GA. Pathophysiology of preeclampsia and the role of serotonin. Eur J Obstet Gynecol Reprod Biol. 2001;95: 12–21. doi:10.1016/S0301-2115(00)00367-5

27. Smith KA, Fairburn CG, Cowen PJ. Relapse of depression after rapid depletion of tryptophan. Lancet. 1997;349: 915–919. doi:10.1016/S0140-6736(05)61726-6

28. Wiciński M, Malinowski B, Puk O, Socha M, Słupski M. Methyldopa as an inductor of

postpartum depression and maternal blues: a review. Biomed Pharmacother. 2020;127: 110196. doi:10.1016/j.biopha.2020.110196

29. Howard LM, Flach C, Mehay A, Sharp D, Tylee A. The prevalence of suicidal ideation identified by the Edinburgh Postnatal Depression Scale in postpartum women in primary care: findings from the RESPOND trial. BMC Pregnancy Childbirth. 2011;11: 57. doi:10.1186/1471-2393-11-57

30. Archchi NSM, Ganegama R, Husna AWF, Chandima DL, Hettigama N, Premadasa J, et al. Suicidal ideation and intentional self-harm in pregnancy as a neglected agenda in maternal health; an experience from rural Sri Lanka. Reprod Health. 2019;16: 166. doi:10.1101/473058

31. Patel V, Rodrigues M, DeSouza N. Gender, poverty, and postnatal depression: a study of mothers in Goa, India. Am J Psychiatry. 2002;159: 43–47. doi:10.1176/appi.ajp.159.1.43

32. Wisner KL, Sit DK, McShea MC, Rizzo DM, Zoretich RA, Hughes CL, et al. Onset timing, thoughts of self-harm, and diagnoses in postpartum women with screen-positive depression findings. JAMA Psychiatry. 2013;70: 490–498. doi:10.1001/jamapsychiatry.2013.87

33. Marecek J. Young Women's Suicide in Sri Lanka: Cultural, ecological, and psychological factors. Asian J Couns. 2006;13: 63–92.

34. Shrestha SD, Pradhan R, Tran TD, Gualano RC, Fisher JRW. Reliability and validity of the Edinburgh Postnatal Depression Scale (EPDS) for detecting perinatal common mental disorders (PCMDs) among women in low-and lower-middle-income countries: a systematic review. BMC Pregnancy Childbirth. 2016;16: 72. doi:10.1186/s12884-016-0859-2

Click here to access/download
Supporting Information
S1 Dataset.sav

~~**Title:** Postpartum depression and associated risk factors among mothers in Galle, Sri Lanka~~

Risk of postpartum depression in Sri Lanka: A population-based study using a validated screening tool

Therese Røysted Solås [1*], Sven Gudmund Hinderaker [1], Lasantha Ubesekara [2], Vijitha De Silva [3]

[1]University of Bergen, Bergen, Norway

[2]Ministry of Health, Southern Province, Sri Lanka

[3]University of Ruhuna, Galle, Sri Lanka

* Corresponding author

E-mail: therese.solas@student.uib.no

Formatted: Not Superscript/ Subscript

Formatted: Space After: 8 pt, Line spacing: Multiple 1.08 li

Formatted: English (United Kingdom)

Authors and affliations

Therese Røysted Solås [1*]

Sven Gudmund Hinderaker [1]

Lasantha Ubesekara [2]

Vijitha De Silva [3]

[1]University of Bergen, Bergen, Norway

[2]Ministry of Health, Southern Province, Sri Lanka

[3]University of Ruhuna, Galle, Sri Lanka

* Corresponding author

E-mail: therese.solas@student.uib.no

Formatted: English (United States)

Abstract

Background: Postpartum depression is an important public health concern. The prevalence of postpartum depression is estimated to be 18~~3~~% worldwide. The purpose of this study was to estimate the prevalence of postpartum depression in Sri Lanka~~a -~~and to investigate its associated risk factors.

Methods: This was a cross-sectional study conducted among 975 mothers in Galle district, Sri Lanka. The prevalence of mothers at risk of postpartum depression was assessed using the Edinburgh Postpartum Depression scale (EPDS) which has been validated for screening for postpartum depression in Sri Lanka with a cut-off score 9 or more. ~~-~~Data from routine records on pregnancy, delivery, postnatal care was collected to investigate possible predictors of postpartum depression. Crosstabulation and multivariable logistic regression was performed to identify risk factors for postpartum depression, defined as EPDS score 9 or more. Prevalence estimates using different cut-offs for postpartum depression was compared to assess the impact of using unvalidated cut-offs for screening.

Results: The prevalence of mothers with EPDS score 9 or more was found to be 9.4% (95%CI: 7.8-11.4); EPDS score 10 or more was 5.6% (95%CI: 4.4-7.3). Postpartum depression (EPDS 9 or more) was associated with the following risk factors: Former history of mental illness (aOR 32.9, 95%CI: 7.9-136.2), high maternal age 30-39 (aOR 2.2, 95%CI: 1.3-3.8), BMI 25.0-29.9 (aOR 2.6, 95%CI: 1.5-4.5), hypertension (aOR 3.6, 95%CI: 1.2-10.9) and newborn death (aOR 28.9, 95%CI: 4.5-185.1). One in five women reported thoughts of self-harm.

Conclusion: A significant proportion of Sri Lankan mothers experience symptoms of postpartum depression, highest risk among mothers who reported former history of mental illness and newborn death. Using a higher cut-off for screening had a substantial impact on the prevalence rate estimates. ~~, but the prevalence is lower than other countries in South Asia.~~ Mothers at increased risk should be identified in antenatal care and is an important target of referral. ~~of postpartum depression should be identified at antenatal clinics and referred when needed.~~

Formatted: Font color: Text 1

55

Introduction

Depression after giving birth is considered a serious public health problem worldwide, and the overall prevalence worldwide is estimated to be 18% [1]. ~~13% [1].~~ Studies suggest that postpartum depression (PPD) not only affects the wellbeing of postpartum women, but also the mother-infant interaction [2]. There is a compelling body of evidence that PPD may influence the social, cognitive and emotional development of the child and infant growth [2].

PPD and its associated risk factors are well studied in high income countries (HICs) but data from low- and middle-income countries (LMICs) is scarce [3]. However, research suggest there is a higher prevalence of PPD in LMICs compared to in HICs [3], which aligns well with low socioeconomic status as a known risk factor [2]. It is widely recognized that PPD is not attributable to one single cause, but rather influenced by complex interactions between environmental, psychosocial and biological risk factors [2]. Several risk factors associated with PPD have been investigated. PPD in mothers is more often observed in those with a former history of mental illness [4]. ~~and with low socioeconomic status[2].~~ Associations with maternal age have shown conflicting results [5]. ~~It is widely recognized that PPD is not attributable to one single cause, but rather influenced by complex interactions between environmental, psychosocial and biological risk factors [2].~~ Furthermore, previous research suggests that thoughts of self-harm in pregnancy is not uncommon [6], as in many populations.

~~PPD and its associated risk factors are well studied in high income countries (HICs) but data from low- and middle-income countries (LMICs) is scarce [5]. However, research suggest there is a higher prevalence of PPD in LMICs compared to in HICs [5], which aligns well with low socioeconomic status as a known risk factor [2]. As poverty is more common in LMICs compared to in HICs, impaired quality of parenting may be associated with higher risks to the child's physical health [7].~~

Screening for and managing PPD is included in *The Global Strategy for women's, children's and adolescent's health (2016-2030)* by the World Health Organization [7]. Several tools to screen or diagnose PPD have been used, and this is reflected in the diversity in reported prevalence in different countries [5]. The Edinburgh Postnatal Depression Scale (EPDS) is a 10-item questionnaire and the most widely used tool globally to screen for mothers at risk of PPD [2]. Each answer is scored 0-3 and a higher sum of scores indicates more symptoms of depression. Originally, the EPDS was developed for screening for PPD in English-speaking women and a cut-off 10 or more for "possible depression" and a cut-off 13 or more for "probable depression" was suggested [8]. Since then, the EPDS has been translated to and validated in many languages using different cut-offs for screening in different countries. Optimal psychometric properties are important for assessing prevalence rates and the overall burden of PPD in countries [9]. In 2006, Matthey et al. put focus on the increasing use of unvalidated EPDS cut-off scores in studies on PPD [9]. Furthermore, he stated that a difference of just 1 point in cut-off score can have a substantial impact on estimates of PPD rates [9].

In South-Asia, the prevalence of PPD have shown to range from 5-49% [1]. Sri Lanka is a lower middle-income country and has been considered a role model for maternal health promotion and for reducing maternal deaths. In Sri Lanka the Sinhala version of the EPDS was introduced and included as a part of postnatal care in 2012 [10] and it has been validated for screening for PPD in mothers with a cut-off 9 or more for depression [11]. In 2011, a cross sectional study representing eight provinces in Sri Lanka estimated the prevalence of PPD to be 27.1% using a cut-off 10 or more [12]. A recent study from 2017 in Galle, Sri Lanka, found a that 7.8% of mothers had an EPDS score 10 or more [13]. To this date there are no published studies available on the prevalence of mothers at risk of PPD in Sri Lanka using the validated cut-off for screening EPDS score 9 or more. Using a higher cut-off may lead to an underestimation of the burden of PPD in Sri Lanka. ~~In Sri Lanka the standard maternal and child care is run by the health system, which includes assessing all postpartum women for depression using the EPDS. A~~

Field Code Changed

Formatted: Not Highlight

~~recent study from 2017 in Galle, Sri Lanka, found a that 7.8% of mothers had an EPDS score 10 or more [9]. This is lower than previously reported in Sri Lanka, like in 2004 in Puttalam district with 32.1% with EPDS score 10 or more [10], and lower than findings from other low and middle-income countries [3].~~ Therefore, in our study we aimed to: 1) repeat this study on EPDS score in ~~pregnant~~ postpartum women at antenatal clinics in Galle using an EPDS cut-off 9 or more 2) evaluate the difference on prevalence estimates using a cut-off 10 or more ~~, and~~3 ~~2~~) investigate risk factors associated with the validated cut-off for screening of PPD of EPDS score 9 or more among pregnant women.

Methods

Study design and study setting

A cross sectional study was conducted among mothers who had delivered from 1st January 2019 to 31st December 2019 in Bope-Poddala, Medical Offices of Health (MOH) Division, Galle district, Sri Lanka. ~~The main outcome measurement was rate of mothers with EPDS score 9 or more.~~ With a population of over 71 000, Bope-Poddala is a semi urban MOH division in Galle district. All three main population groups of Sri Lanka live there: Sinhalese, Tamils and Muslims. Here preventive care services are provided through 20 divisions of the MOH. ~~, and around one thousand births take place each calendar year.~~ Antenatal and postnatal care is provided to the mothers through five maternal and child health field clinics. In Sri Lanka the antenatal care attendance is very high, and nearly 100% of deliveries occur in health facilities. Maternal mental health services are provided during regular visits in the clinics by a medical officer of mental health.

Participants

In Bope-Poddala, around one thousand births take place each calendar year. The sample size for the postpartum mothers to be included in the study was estimated using OpenEpi.com calculator [14]. ~~We assumed that the~~Assuming that the prevalence of PPD in Galle would be around 18% [1] ~~13% [4]~~ and ~~aimed~~ aiming at estimating the proportion with a precision of +/- 2.5%, t~~. T~~he required sample size for our study was estimated to be n=~~695~~907. Therefore, all the postpartum mothers who delivered from 1st January 2019 to 31st December 2019 ~~during 2019 would be a sufficient sample for this study~~were included in the study. Exclusion criteria was incomplete EPDS records.

~~All mothers had complete EPDS records and no mothers were excluded.~~

Study tool

In Sri Lanka, all mothers are routinely screened once at the clinics for PPD 4 to 6 weeks after delivery using Sinhala or Tamil version of Edinburgh Postpartum Depression Scale (EPDS).

Formatted: Space After: 8 pt

Formatted: Font: (Default) +Body (Calibri)

~~Data collection~~

~~All mothers were routinely screened for PPD 4 to 6 weeks after delivery using Sinhala or Tamil version of Edinburgh Postpartum Depression Scale (EPDS). The EPDS consists of 10 questions pertaining to depression, and each answer is scored 0-3; a higher sum of scores indicates more symptoms of depression.~~ An EPDS score 9 or more for depression has been validated for screening for PPD with the Sinhala version of the EPDS in Sri Lanka, with a sensitivity and specificity of 89.9% and 78.9%, respectively [11]. The Tamil version of the EPDS is used for screening in the Tamil population. The Tamil version of the EPDS has been validated for screening in Tamil Nadu, India using the same values for depression, EPDS 9 or more, with a ~~specificity~~ sensitivity of 94.1% and a specificity of 90.2% [15]. The EPDS question 10 is an item on thoughts of self-harm.

In current practice, ~~T~~the EPDS ~~was completed~~is filled on paper forms by the mothers at their postnatal visit at the clinic and then collected and kept by the midwives. If the mother ~~was~~ cannot read Tamil or Sinhala ~~illiterate~~ the EPDS ~~would~~ is~~be~~ completed by the midwife through interview with the mother. Mothers with an EPDS score 9 or more ~~were~~ are referred to a medical officer of health at the clinics who then refer~~red~~ the mother to their designated medical officer of mental health (MOMH). MOMH manage some of the cases, and if necessary, refers the mother to psychiatrist care at the nearest hospital. In severe cases the medical officer of health refers directly to a consultant psychiatrist. MOMHs and psychiatrist diagnose mothers with PPD using the ICD-10 classification for diagnosis, usually through clinical interview with the mother.

Data collection

All pregnant women have EPDS records and routine records on pregnancy, delivery, postnatal care in the B part of the pregnancy record which is kept in the clinics. For our study, ~~The~~ determinants were collected as quantitative clinical data from these records into a data extraction sheet by a trained research assistant. The data extraction sheet included sociodemographic, obstetric and baby related variables. The collected data was translated to English and double-entered into the electronic database EpiData 3.1. After ensuring that data from various sources were linked to the same person, the names and identifiers were removed. In Sri Lanka, pregnancy outside marriage is very rare and thus there is no place for marital status in the pregnancy record. Local midwives had a way of knowing, but could not identify anyone unmarried in the study participants. However, this is associated with uncertainty.

Formatted: Font: 16 pt, Bold, Font color: Text 1

Formatted: Line spacing: Double, Pattern: Clear (Background 1)

Formatted: Font: (Default) +Body (Calibri), Font color: Text 1

Formatted: Space After: 8 pt, Pattern: Clear (Background 1)

Formatted: Font: Cambria

Formatted: Space After: 8 pt

Outcome measures and exposure variables

The main outcome measurement was rate of mothers with EPDS score 9 or more. Exposure variables were: maternal age (under 20, 20-29, 30-39 and 40 and more); educational level (completed secondary school ordinary level or below, completed above secondary school ordinary level); occupation (housewife or non-housewife); socioeconomic status (calculated according to the occupation of the mother and the husband, and categorized into SE 1-2 and SE 3-5); number of living children (0, 1, 2-3 and >4); former history of mental illness (yes/no); maternal Body Mass Index (BMI) (less than 18.5, 18.5-24.9, 25.0-29.9 and 30 and more); hypertension during pregnancy (yes/no); diabetes during pregnancy (yes/no); history of subfertility (yes/no); death of newborn defined as death of liveborn within 28 days (yes/no); gender of the baby (female or male); congenital abnormalities (yes/no); birthweight (less than 2500g, 2500g or more); establishment of breastfeeding (yes/no); other complications from delivery notes (yes/no).

Data analysis

The database was exported into IBM SPSS 26 version for statistical analysis. ~~The collected data was translated to English and double-entered into the electronic database EpiData 3.1 and then exported into IBM SPSS 26 version for statistical analysis.~~ The prevalence of mothers at risk of PPD was calculated as percentages of mothers with an EPDS score 9 or more. Differences in categorical variables according to the prevalence of EPDS score 9 or more were analyzed with Pearson's Chi-square test (corrected by Fisher exact test for small samples) and level of significance was set at $p<0.05$. One-way ANOVA was performed to compare the mean EPDS score for each independent variable. Possible predictors of PPD were examined in univariate and multivariable models. In the final model we adjusted with maternal age, BMI, former history of mental illness, hypertension, history of subfertility and newborn death. Odds ratios (OR) and

Formatted: Font: 16 pt, Bold, Font color: Auto
Formatted: Line spacing: Double
Formatted: Font: Cambria
Formatted: Space After: 8 pt
Formatted: Font: Cambria
Formatted: Font: Cambria
Formatted: Font: 16 pt, Bold

adjusted Odds ratios (aOR) with 95% confidence intervals (95%CI) were calculated. The prevalence of mothers reporting thoughts of self-harm was calculated as percentages of mothers reporting “never”, “hardly ever”, “sometimes” and “many times” on the EPDS item 10.

Ethical issues

Formatted: English (United States)

Ethical clearance for the present study was obtained by Ethics Review Committee, Faculty of Medicine, University of Ruhuna and by REK, Regionale komiteer for medisinsk og helsefaglig forskningsetikk. Informed consent was not required as ~~this short questionnaire~~the EPDS was used as routine screening service and introduced into antenatal clinics several years before. The data archive had no names or identifiers.

Results

Included in the study were 975 pregnant women. All mothers had complete EPDS records and no mothers were excluded. Table 2 shows the characteristics of the mothers. Among the respondents the mean age was 29 years, ranging from 16 to 45 years. The majority of the mothers (70.5%) were housewives, and half of the mothers (48.1%) had completed education above ordinary level examination secondary school. Former history of mental illness was identified in 12 women (1.2%).

Table 1 shows the EPDS scores among the 975 women studied; 9.4% (n=92) had an EPDS score 9 or more; and 5.6% (n=55) had a EPDS score 10 or more. An EPDS score 12 or more was seen in 2.1% (n=20) of the participants.

Formatted: Space After: 0 pt, Line spacing: Double, Pattern: Clear (Background 1)

Table 1. Frequency distribution of the EPDS scores with sensitivity analysis among mothers in Galle, 2019

EPDS	Frequency (n)	Percent (%)	95%CI
Total	975	100	-
EPDS score 9 or more	92	9.4	7.8-11.4
EPDS score 10 or more	55	5.6	4.4-7.3
EPDS score 11 or more	29	3.0	2.1-4.2
EPDS score 12 or more	20	2.1	1.3-3.1

Association of postpartum depression with risk factors

Table 2 shows mean EPDS score by exposure variables and corresponding logistic regression analysis of these determinants of PPD, defined as EPDS score 9 or more. Compared to mothers aged 20-29, those aged 30-39 had twice the risk of PPD (aOR 2.2, 95%CI: 1.3-3.8). Mothers who had experienced newborn death had a higher risk of PPD (aOR 28.9, 95%CI: 4.5-185.1), and mothers who reported a former history of mental illness had a highly increased risk of PPD (aOR 32.9, 95%CI: 7.9-136.2). Mothers with a BMI <18.5 (aOR 3.0, 95%CI: 1.4-6.3) and BMI 25.0-29.9 (aOR 2.6, 95%CI: 1.5-4.5) had a higher risk of PPD than those with normal BMI. Those who had hypertension were 3.6 times more likely (aOR 3.6, 95%CI: 1.2-10.9) to develop PPD as compared to mothers without hypertension.

No significant difference was found between the occurrence of PPD and obstetric factors. Baby gender and late breast-feeding initiation was not significantly associated with the development of PPD. PPD defined as EPDS score 10 or more showed similar associations with the same variables (not included in the table).

Table 2. Determinants of postpartum depression (EPDS score 9 or more) among mothers in Galle, 2019~~, analysed by ANOVA with mean EPDS score, and multivariable logistic regression with odds ratios~~

Variables	Total	EPDS 9 or more	Mean EPDS (95% CI)	OR (95% CI)	aOR (95% CI)
Maternal age					
≤19	52	2	4.6 (4.0-5.2)	0.6 (0.1-2.6)	0.8 (0.2-3.7)
20-29	459	29	3.8 (3.6-4.1)	1	1
30-39	447	59	**4.6 (4.3-4.9)**	**2.3 (1.4-3.6)**	**2.2 (1.3-3.8)**
≥40	17	2	4.2 (2.6-5.9)	2.0 (0.4-9.0)	1.0 (0.1-8.6)
Education					
≤Ordinary level	505	46	4.4 (4.1-4.6)	1	1
>Ordinary level	469	46	4.1 (3.8-4.4)	1.1 (0.7-1.7)	1.1 (0.6-1.7)
Missing	1				
Occupation					
Housewife	687	68	4.3 (4.1-4.6)	1	1
Non-housewife	288	24	4.0 (3.7-4.3)	0.8 (0.5-1.3)	0.7 (0.4-1.2)
Socioeconomic class					
1-2	194	13	3.6 (3.3-4.0)	0.6 (0.3-1.2)	0.6 (0.3-1.2)
3-5	781	79	4.4 (4.2-4.6)	1	1
Number of living children					
0	0	0	--	-	-
1	428	34	4.1 (3.8-4.4)	1	1
2-3	489	52	4.3 (4.0-4.5)	1.4 (0.9-2.2)	0.8 (0.4-1.3)
>4	57	6	4.5 (3.9-5.2)	1.4 (0.5-3.4)	0.5 (0.2-1.4)
Missing	1				

History of mental illness					
Yes	12	9	**11.0 (8.1-13.9)**	**31.8 (8.4-119.8)**	**32.9 (7.9-136.2)**
No	963	83	4.1 (4.0-4.3)	1	1
BMI					
≤18.4	119	13	**4.4 (3.8-5.0)**	**2.0 (1.0-4.0)**	**3.0 (1.4-6.3)**
18.5-24.9	462	27	4.0 (3.7-4.2)	1	1
25.0-29.9	290	43	**4.6 (4.2-4.9)**	**2.8 (1.7-4.7)**	**2.6 (1.5-4.5)**
≥30	57	3	4.1 (3.4-4.9)	0.9 (0.3-3.1)	0.8 (0.2-2.8)
Missing	47				
Hypertension					
**Yes**	17	6	**6.3 (4.4-8.1)**	**5.5 (2.0-15.3)**	**3.6 (1.2-10.9)**
No	958	86	4.2 (4.0-4.4)	1	1
Diabetes					
Yes	54	8	5.0 (4.0-5.9)	1.7 (0.8-3.8)	0.7 (0.2-1.9)
No	921	84	4.2 (4.0-4.4)	1	1
History of subfertility					
Yes	39	7	5.3 (4.0-6.5)	2.2 (0.9-5.1)	1.0 (0.4-2.8)
No	936	85	4.2 (4.0-4.4)	1	1
Newborn death					
Yes	6	4	**9.0 (4.6-13.4)**	**20.0 (3.6-110.9)**	**28.9 (4.5-185.1)**
No	969	88	4.2 (4.0-4.4)	1	1
Baby gender					
Male	487	41	4.2 (3.9-4.4)	0.8 (0.5-1.2)	0.7 (0.4-1.2)
Female	487	51	4.3 (4.0-4.6)	1	1

Missing	1				
Congenital abnormalities					
Yes	8	2	6.0 (2.1-9.9)	3.2 (0.6-16.3)	1.3 (0.1-11.8)
No	966	90	4.2 (4.0-4.4)	1	1
Missing	1				
Birthweight					
<2500	89	6	4.3 (3.7-5.0)	0.7 (0.3-1.6)	0.5 (0.2-1.3)
≥2500	886	86	4.2 (4.0-4.4)	1	1
Breast feeding					
Yes	429	35	4.2 (4.0-4.5)	0.8 (0.5-1.2)	0.6 (0.4-1.1)
No	546	57	4.2 (4.0-4.5)	1	1
Delivery complication					
Yes	40	4	4.6 (3.4-5.8)	1.1 (0.4-3.1)	0.5 (0.2-2.0)
No	934	88	4.2 (4.0-4.4)	1	1
Missing	1				
Baby birth complication					
Yes	23	2	5.0 (3.4-6.5)	0.9 (0.2-3.9)	0.1 (0.0-1.4)
No	936	90	4.2 (4.0-4.4)	1	1
Missing	16				
Malpresentation					
Yes	45	1	4.1 (3.3-5.0)	0.2 (0.0-1.5)	0.1 (0.0-1.3)
No	929	91	4.2 (4.0-4.4)	1	1
Missing	1				

** Analysed by ANOVA with mean EPDS score, and multivariable logistic regression with odds ratios.*

Regression analysis included these variables in the adjustment model: maternal age, BMI, former history of mental illness, hypertension, history of subfertility and newborn death. Other variables

Formatted: Font: Italic

Formatted: Font: Not Bold, Italic

were adjusted with the same model.

Thoughts of self-harm

Table 3 shows responses to EPDS Question 10 and the associated mean total EPDS. Nearly 1 out of 5 (n=188) study participants reported "the thought of harming myself has occurred to me". Out of those 188 participants, 26.1% had an EPDS score 9 or more. We see that the more frequent such thoughts are reported, the more likely depression occurred.

Table 3. EPDS question 10: "The thought of harming myself has occurred to me"

Response	Frequency (n)	Percent (%)	Mean total EPDS (95%CI)
Total	*975*	*100*	-
Never	*787*	*80.7*	3.6 (3.4-3.8)
Hardly ever	*174*	*17.8*	6.6 (6.2-7.0)
Sometimes	*14*	*1.4*	8.3 (7.0-9.6)
Many times	*0*	*0*	-

Discussion

In this study in Sri Lanka among 975 women who ~~just had given birth~~had given birth between 4 to 6 weeks ago we found that 9.4% (95%CI: 7.8-11.4) had an EPDS score 9 or more, which means they are referred to mental health services for further management and diagnosis of PPD. The factor that showed the strongest association with PPD was former history of mental illness, and this was not investigated in the former publication. ~~In 2017 the results of a similar study reported that 7.8% mothers had an EPDS score 10 or more, compared to 5.6% (95%CI: 4.4-7.3) in our material which is significantly lower than the 2017 results [9]. The factor that showed the strongest association with PPD was former history of mental illness.~~ Several maternal

characteristics and factors were associated with PPD: high maternal age, overweight, underweight, hypertension and newborn death.

Various studies on PPD in Sri Lanka have shown a wide variation in prevalence, perhaps partly due to differences in methods. In 2017 a similar study conducted in Galle, Sri Lanka reported that 7.8% mothers had an EPDS score 10 or more [13], compared to 5.6% (95%CI: 4.4-7.3) in our material. Our findings support the previous finding of low risk of PPD in Galle, Sri Lanka. However, we see that when measuring the prevalence rates of PPD using a EPDS cut-off 9 or more compared to a cut-off 10 or more a difference of 3.8% in prevalence estimates occurred. This supports the statement by Matthey et. al that a difference of just 1 point in cut-off score can have a substantial impact on postpartum depression rates and highlights the importance of using the validated cut-off for screening in Sri Lanka [9]. On human development index, Galle ranked between mid and top on the health index and high on education index in Sri Lanka in 2013 [16] which may contribute to the low prevalence. Furthermore, the prevalence is lower than ~~the~~ findings in other countries in South-Asia. A validation study from Nepal showed a prevalence of PPD at 17.1%. It defined depression as EPDS 13 or more and had a sensitivity of 92% and a specificity of 95.6% [17]. A meta-analysis from India estimated a pooled prevalence of PPD at 22% [18]~~Another study from India showed a prevalence of 24% defining depression as EPDS 12 or more, and showed a sensitivity of 92% and specificity of 85% [16]~~. Compared to the neighboring countries, Sri Lanka have made more progress on maternal health and child mortality indicators, as well as other indicators such as GDP per capita and education [19].

In our study, mothers aged 30-39 had twice the risk of developing depressive symptoms compared to those aged 20-29. The same findings are reported in the paper from the same place in 2017 [13]. A higher percentage of EPDS score 9 or more in the age group 30-39 compared to other age groups was observed in both primiparous mothers and in multiparous mothers. Other sociodemographic risk factors such as educational level and being a housewife did not show

significant associations with PPD.

In terms of maternal BMI, underweight women (BMI<18.5) had a three times risk of PPD compared to women of normal weight, which is consistent with findings in Nepal [20] and Sweden [21]. It is widely recognized that BMI is likely to be influenced by income [16] and adjusting for only socioeconomic class, the association between underweight and PPD was no longer significant. In Sri Lanka, overweight (BMI 25.0-29.9) is far more prevalent than underweight and was in our study associated with more than twice the risk of developing PPD compared to mothers with normal bodyweight. ~~Studies~~ One study suggest that overweight in Sri Lanka is associated with urban living and being in middle age [22].

We found that hypertension was associated with increased risk of PPD. In our study, the mothers with hypertension were more often of advanced age, which reflects the prevalence of hypertension in the general population [23]. Also, hypertensive mothers had more gestational diabetes, also increasing with age. The possible relationship between preeclampsia and PPD has previously been described in studies from South Korea [24] and Netherlands [25]. In pre-eclampsia, the blood level of serotonin is known to increase [26] and previous studies have hypothesized that this may be linked to a decreased level of serotonin in the brain [25]. A possible role of alterations in serotonin function has been noted in the pathophysiology of general depression [27]. A further complication may be that hypertension in pregnancy is commonly treated with methyldopa, of which a known side effect is depressive symptoms [28]. However, hypertension and preeclampsia could not be differentiated from the pregnancy record in our study.

Thoughts of self-harm was reported by almost 20%, of whom the vast majority responded “hardly ever”. In a randomized controlled trial in the UK, it was found that the response “hardly ever” on EPDS item 10 in mothers was not concordant with suicidality as measured by a clinical

interview [29]. A study published in 2019 which included 475 pregnant women in Anuradhapura, Sri Lanka reported rates on thoughts of self-harm at 5.9% including "hardly ever" [30], and in Goa, India, it was 14.3% [31],[6]. In Pittsburgh, USA, with a study population of 10 000 mothers, the observed rate on thoughts of self-harm 4 to 6 weeks postpartum was 3.2%, out of whom the majority had screen-positive findings on depression [32]. This is in contrast to our study, where only 1 out of 4 women with thoughts about self-harm had in addition an EPDS score 9 or more; however, we noticed that the more frequent the mothers' thoughts were, the more signs of depression occurred, shown by increasing mean EPDS score. In Sri Lanka, mothers are usually not referred to a psychiatrist based on the response on EPDS item 10. However, research on self-harm behavior in Sri Lanka is limited and it remains to be shown whether self-harm ideation as measured by the EPDS item 10 can predict physical self-harm in Sri Lankan mothers.

In our study mothers who had experienced death of a newborn more often had EPDS score 9 or more, although grief reactions may be difficult to distinguish from PPD symptoms. We also found that former history of mental illness predicts PPD, which is consistent with the findings by O'Hara and Swain [4]. In our study, a diagnosis of mental illness before the pregnancy was included but not further defined. However, the number of mothers identified (n=12) may not represent the total number of mothers who ever had a mental illness; perhaps those with a more recent diagnosis were more likely to report on this item but we do not have this information. In Sri Lanka, stigmatizing beliefs on mental health have been reported to affect help-seeking behavior [33]. As in many Asian cultures, emotional restraint and self-control are perceived as desirable qualities in women, and to seek counselling for mental health issues may be considered unacceptable by family members [33]. Our findings suggest that attendants reporting a past mental illness may be considered at risk of PPD, and may benefit from referral.

Our study had several strengths. The adequate sample size provides some confidence to the precision in our results, and missing data was minimal. The transfer of information in paper forms into digital data was validated by double data entry. We think the study gives a good representation of the situation for pregnant women in the area, as almost all pregnant women there attend ANC. Furthermore, our study is the first study since the EPDS was included as a part of postnatal care in 2012 to use the validated cut-off for screening when estimating mothers at risk of PPD in Sri Lanka.

The study also had some limitations. The cross-sectional study design can only analyze associations and not causation, but this is in line with our aims. Recall is generally associated with some uncertainty, especially about feelings in the past, but limiting to the past week may provide more credibility to the findings. Misinterpretation of the questions and social taboos may have led to response bias, but the local author did not see such obvious mistakes. The cut-off threshold for categorizing depression is a matter of discussion; we used a validated cut-off for screening for PPD in Sri Lanka, and we also tested the association with other cut-offs as well (Table 1) and found similar associations (not shown in table). The tool we used was tested and validated in both Sinhala and Tamil language. Nonetheless, great caution must be taken when comparing the results of EPDS screening for PPD in different countries as there is no international standard for how to translate, nor validate, the EPDS in local languages [34].

Conclusion

The prevalence of EPDS score 9 or more, indicating risk of PPD, in Galle, Sri Lanka was estimated to be 9.4% (95%CI: 7.8-11.4). ~~The prevalence of EPDS score 9 or more, indicating risk of PPD, in Galle, Sri Lanka was estimated to be 9.4% (95%CI: 7.8-11.4). This is lower than other countries in South Asia~~A higher cut-off for screening had a substantial impact on the prevalence rate estimates and thus highlights the importance of using the validated cut-off EPDS score 9 or more

for future studies on PPD in Sri Lanka. The major predisposing factors for postpartum depression were former history of mental illness, high maternal age, overweight, hypertension and newborn death. Furthermore, the number of mothers reporting thoughts of self-harm was high. Mothers at increased risk of PPD is an important target of referral to specialist.

Aknowledgements

The authors would like to thank all Public Health midwives of Bope-Poddala MOH division for their effort and cooperation in collecting data for this study, as well as our research assistant Ms. Prabodha Lakmali. We would like to thank Prof. Ganesh Acharya for his insightful suggestions and reading of the manuscript. This research was supported by University of Ruhuna, Sri Lanka and University of Bergen, Norway.

References

1. Hahn-Holbrook J, Cornwell-Hinrichs T, Anaya I. Economic and Health Predictors of National Postpartum Depression Prevalence: A Systematic Review, Meta-analysis, and Meta-Regression of 291 Studies from 56 Countries. Front Psychiatry. 2018;8. doi:10.3389/fpsyt.2017.00248
2. Stewart DE, Robertson E, Dennis C-L, Grace SL, Wallington T. Postpartum depression: Literature review of risk factors and interventions. Toronto: University Health Network Women's Health Program for Toronto Public Health. 2003.
3. Fisher J, Cabral de Mello M, Patel V, Rahman A, Tran T, Holton S, et al. Prevalence and determinants of common perinatal mental disorders in women in low- and lower-middle-income countries: a systematic review. Bull World Health Organ. 2012;90: 139–149. doi:10.2471/BLT.11.091850
4. O'Hara MW, Swain AM. Rates and risk of postpartum depression-a meta-analysis. Int Rev Psychiatry. 1996;8: 37–54. doi:10.3109/09540269609037816
5. Norhayati MN, Hazlina NH, Asrenee AR, Emelin WM. Magnitude and risk factors for postpartum symptoms: a literature review. J Affect Disord. 2015;175: 34–52. doi:10.1016/j.jad.2014.12.041
6. Lindahl V, Pearson JL, Colpe L. Prevalence of suicidality during pregnancy and the postpartum. Arch Womens Ment Health. 2005;8: 77–87. doi:10.1007/s00737-005-0080-1
7. Kuruvilla S, Bustreo F, Kuo T, Mishra CK, Taylor K, Fogstad H, et al. The global strategy for women's, children's and adolescents' health (2016-2030): A roadmap based on evidence and country experience. Bull World Health Organ. 2016;94: 398–400. doi:10.1016/b978-0-444-89007-8.50010-1
8. Cox J, Holden J, Sagovsky R. Detection of Postnatal Depression: Development of the 10-item Edinburgh Postnatal Depression Scale. BJPsych. 1987;150: 782-786. doi:10.1192/bjp.150.6.782.
9. Matthey S, Henshaw C, Elliott S, Barnett B. Variability in use of cut-off scores and formats

Formatted: English (United States)

on the Edinburgh Postnatal Depression Scale - Implications for clinical and research practice. Arch Womens Ment Health. 2006;9: 309–315. doi:10.1007/s00737-006-0152-x

10. Agampodi SB, Agampodi TC. Antenatal Depression in Anuradhapura, Sri Lanka and the Factor Structure of the Sinhalese Version of Edinburgh Post Partum Depression Scale among Pregnant Women. PLoS One. 2013;8. doi:10.1371/journal.pone.0069708

11. Rowel D, Jayawardena P, Fernando N. Validation of the Sinhala translation of Edinburgh Postnatal Depression Scale. Ceylon Med J. 2008;53: 10–13. doi:10.4038/cmj.v53i1.219

12. Agampodi TC, Agampodi SB, Wickramasinghe WA, Adhikari AM, Chathurani HK. Post partum depression - a problem that needs urgent attention. Ceylon Med J. 2011;56: 183–184. doi:10.4038/cmj.v56i4.3907

13. Fan Q, Long Q, De Silva V, Gunarathna N, Jayathilaka U, Dabrera T, et al. Prevalence and risk factors for postpartum depression in Sri Lanka: A population-based study. Asian J Psychiatr. 2020;47: 101855. doi:10.1016/j.ajp.2019.101855

14. Dean AG, Sullivan KM, Soe MM. OpenEpi: Open Source Epidemiologic Statistics for Public Health, Version [cited 17 May 2021]. Available from: www.OpenEpi.com, updated 6 April 2013.

15. Benjamin D, Chandramohan A, Annie IK, Prasad J, Jacob KS. Validation of the Tamil version of Edinburgh post-partum depression scale. J Obstet Gynecol India. 2005;55: 241–243.

16. United Nations Development Programme. Sri Lanka Human Development report 2012. UNDP Sri Lanka, 2012.

17. Bhusal BR, Bhandari N, Chapagai M, Gavidia T. Validating the Edinburgh Postnatal Depression Scale as a screening tool for postpartum depression in Kathmandu, Nepal. Int J Ment Health Syst. 2016;10: 71. doi:10.1186/s13033-016-0102-6

18. Upadhyay RP, Chowdhury R, Salehi A, Sarkar K, Singh SK, Sinha B, et al. Postpartum depression in India: a systematic review and meta-analysis. Bull World Health Organ. 2017;95: 706-717B. doi:http://dx.doi.org/10.2471/BLT.17.192237

19. The World Bank. World Development Indicators, The World Bank Group, 2020 [cited 12 May 2021]. Available from: https://www.worldbank.org/en/home.

20. Ho-Yen SD, Bondevik GT, Eberhard-Gran M, Bjorvatn B. Factors associated with depressive symptoms among postnatal women in Nepal. Acta Obstet Gynecol Scand. 2007;86: 291–297. doi:10.1080/00016340601110812

21. Silverman ME, Smith L, Lichtenstein P, Reichenberg A, Sandin S. The association between body mass index and postpartum depression: a population-based study. J Affect Disord. 2018;240: 193–198. doi:10.1016/j.jad.2018.07.063

22. Katulanda P, Jayawardena MAR, Sheriff MHR, Constantine GR, Matthews DR. Prevalence of overweight and obesity in Sri Lankan adults. Obes Rev. 2010;11: 751–756. doi:10.1111/j.1467-789X.2010.00746.x

23. Katulanda P, Ranasinghe P, Jayawardena R, Constantine GR, Rezvi Sheriff MH, Matthews DR. The prevalence, predictors and associations of hypertension in Sri Lanka: a cross-sectional population based national survey. Clin Exp Hypertens. 2014;36: 484–491. doi:10.3109/10641963.2013.863321

24. Youn H, Lee S, Han SW, Kim LY, Lee TS, Oh MJ, et al. Obstetric risk factors for depression during the postpartum period in South Korea: a nationwide study. J Psychosom Res. 2017;102: 15–20. doi:10.1016/j.jpsychores.2017.09.003

25. Blom EA, Jansen PW, Verhulst FC, Hofman A, Raat H, Jaddoe VWV, et al. Perinatal complications increase the risk of postpartum depression. The Generation R Study. BJOG. 2010;117: 1390–1398. doi:10.1111/j.1471-0528.2010.02660.x

26. Bolte AC, van Geijn HP, Dekker GA. Pathophysiology of preeclampsia and the role of serotonin. Eur J Obstet Gynecol Reprod Biol. 2001;95: 12–21. doi:10.1016/S0301-2115(00)00367-5

27. Smith KA, Fairburn CG, Cowen PJ. Relapse of depression after rapid depletion of tryptophan. Lancet. 1997;349: 915–919. doi:10.1016/S0140-6736(05)61726-6

28. Wiciński M, Malinowski B, Puk O, Socha M, Słupski M. Methyldopa as an inductor of

Formatted: English (United States)

postpartum depression and maternal blues: a review. Biomed Pharmacother. 2020;127: 110196. doi:10.1016/j.biopha.2020.110196

29. Howard LM, Flach C, Mehay A, Sharp D, Tylee A. The prevalence of suicidal ideation identified by the Edinburgh Postnatal Depression Scale in postpartum women in primary care: findings from the RESPOND trial. BMC Pregnancy Childbirth. 2011;11: 57. doi:10.1186/1471-2393-11-57

30. Archchi NSM, Ganegama R, Husna AWF, Chandima DL, Hettigama N, Premadasa J, et al. Suicidal ideation and intentional self-harm in pregnancy as a neglected agenda in maternal health; an experience from rural Sri Lanka. Reprod Health. 2019;16: 166. doi:10.1101/473058

31. Patel V, Rodrigues M, DeSouza N. Gender, poverty, and postnatal depression: a study of mothers in Goa, India. Am J Psychiatry. 2002;159: 43–47. doi:10.1176/appi.ajp.159.1.43

32. Wisner KL, Sit DK, McShea MC, Rizzo DM, Zoretich RA, Hughes CL, et al. Onset timing, thoughts of self-harm, and diagnoses in postpartum women with screen-positive depression findings. JAMA Psychiatry. 2013;70: 490–498. doi:10.1001/jamapsychiatry.2013.87

33. Marecek J. Young Women's Suicide in Sri Lanka: Cultural, ecological, and psychological factors. Asian J Couns. 2006;13: 63–92.

34. Shrestha SD, Pradhan R, Tran TD, Gualano RC, Fisher JRW. Reliability and validity of the Edinburgh Postnatal Depression Scale (EPDS) for detecting perinatal common mental disorders (PCMDs) among women in low-and lower-middle-income countries: a systematic review. BMC Pregnancy Childbirth. 2016;16: 72. doi:10.1186/s12884-016-0859-2

EDITOR

Please ensure that your manuscript meets PLOS ONE's style requirements, including those for file naming. The PLOS ONE style templates can be found at https://journals.plos.org/plosone/s/file?id=wjVg/PLOSOne_formatting_sample_main_body.pdf and https://journals.plos.org/plosone/s/file?id=ba62/PLOSOne_formatting_sample_title_authors_affiliations.pdf

We note that you have indicated that data from this study are available upon request. PLOS only allows data to be available upon request if there are legal or ethical restrictions on sharing data publicly. For more information on unacceptable data access restrictions, please see http://journals.plos.org/plosone/s/data-availability#loc-unacceptable-data-access-restrictions.

In your revised cover letter, please address the following prompts:

a) *If there are ethical or legal restrictions on sharing a de-identified data set, please explain them in detail (e.g., data contain potentially sensitive information, data are owned by a third-party organization, etc.) and who has imposed them (e.g., an ethics committee). Please also provide contact information for a data access committee, ethics committee, or other institutional body to which data requests may be sent.*

b) *If there are no restrictions, please upload the minimal anonymized data set necessary to replicate your study findings as either Supporting Information files or to a stable, public repository and provide us with the relevant URLs, DOIs, or accession numbers. For a list of acceptable repositories, please see http://journals.plos.org/plosone/s/data-availability#loc-recommended-repositories.*

Additional Editor Comments:

Comment 1: *The title of the current paper and that by Fan et al., 2020 are quite similar. Could you please come out clearly as to what differs between your paper and the earlier published one, more so when the study population seems to be the same*
Response: Thank you for pointing this out. In the revised version we have chosen to highlight that this study uses the validated cut-off for screening for PPD. The title is changed to "Risk of postpartum depression in Sri Lanka: A population-based study using a validated screening tool".

Comment 2: *Lines 17 & 40: The prevalence estimates provided here were 1996 figures... could there not have been much more recent ones? Also add the estimates from South Asia - as you compare it in the conclusion*
Response: Good point. We have changed the reference on prevalence estimates in line

16 and line 104 to a more recent one by Hahn-Holbrook (2018). We also added prevalence ranges of PPD in South Asia in the introduction in line 73. However, we removed the sentence "this is lower than other countries in South Asia" in the conclusion as we have put more focus to screening for PPD in Sri Lanka in the revised version.

Comment 3: *Lines 56 - 58: I am not clear about this point... what has this to do with PPD? I read over the cited article, did not come across a relationship between poverty and quality of parenting... please clarify...*
Response: We have deleted this sentence.

Comment 4: *Lines 92 - 93: Why the assumption based on 1996 figures? More so, when there was a 2017 study in the same population?*
Response: At the time of planning and estimating our sample size we did not have the 2017 figures. As we knew that around 1000 births take place each calendar year in Galle, we estimated that in our sample 1000 mothers would give a reasonable precision and power. We calculated samples size both using the 1996 figures and using lower hypothetical figures with not related references. Basically our considerations tested more but only referred to the published old number. As we have now changed the 1996 reference in the introduction to a more recent one, we also updated the sample size estimation reference to be 18% Hahn-Holbrook (2018).

Comment 5: *Lines 94 - 95: Why all mothers????? We the readers are not privy to information on deliveries in the study community... Also, the results section (line 159) has 975 persons... you have not clearly stated how was that arrived at... were those all the mothers interviewed? any mothers not included? response rate?*
Response: Thank you for pointing this out. Basically all women who attended clinic after delivery are asked to fill the EPDS questionnaire screening them for postpartum depression. It is part of the routine public services at Galle, and we do not know whether any of them refused to fill the form, as we have only the filled forms. Exclusion criteria was incomplete EPDS records. We adjusted the methods section according to you recommendation, and added a line in the first paragraph in results section in line 177 to make this clearer.

Comment 6: *Line 98: Will suggest this section be structured as e.g., data collection methods, tools, and process.*
Response: Thank you for this great suggestion. We have now made the following subsections under methods "study design and study setting", "participants", "study tool", "data collection", "outcome measures and exposure variables", "data analysis" and "ethical issues".

Comment 7: *Lines 106 - 107: check this.... specificity????*
Response: Thank you for notifying this mistake. It has now been corrected in line 117-

118.

Comment 8: *Lines 109 - 115: Is the information presented here related to data collection?*
Response: No, the information presented was related to the current practice on screening for PPD in Sri Lanka. Thank you for notifying this. We have now divided the methods section into more subheadings to separate our data collection from the study tool according to your recommendation.

Comment 9: *Line 114: Is the ICD used for classification or diagnosis?*
Response: Yes, we have now changed the line 127 to be more precise.

Comment 10: *Line 126: Label this as a sub-section e.g., variables considered.*
Response: Thank you for this recommendation, we agree to add a subsection before this paragraph and have adjusted it accordingly.

Comment 11: *Lines 159 - 162: Please make reference to table 2.*
Response: Table 2 is now referred to in the text line 178.

Comment 12: *Lines 193 - 195: Revise the title to be much more concise.*
Response: We have shortened the title of table 2.

Comment 13: *Line 201: Mention "EPDS Question 10" in the "Methods section" as part of the data collection tools.*
Response: Thank you for this recommendation. We added this information under methods section in line 118.

Comment 14: *Lines 214 - 216: Were these characteristics also in the 2017 study? If so, state that clearly... if not, then provide the appropriate citation.*
Response: Thank you, this needed clarification. The factors in our study that showed the strongest association with PPD was former history of mental illness and death of the newborn, both not mentioned in the 2017 study. Hypertension was associated with PPD and not studied separately in the former study. High maternal age was associated with PPD in the former study, and this is mentioned in maternal age section in discussion.

Comment 15: *Finally, please follow the reporting guidelines for Cross-sectional studies as provided in the link to guide your paper: https://www.equator-network.org/reporting-guidelines/strobe/. Also review the PLOS ONE guidelines on publications: https://journals.plos.org/plosone/s/criteria-for-publication*
Response: Thank you for providing us with this information. We will adjust the paper according to the guidelines provided.

REVIEWER

Reviewer #1: The study addresses a key issue however there are some modifications required.

Background

Comment 16: *The authors provide some useful information about postpartum depression in the background. Considering that a very similar study had already been conducted in the catchment area however, I think the authors could have provided more information on PPD in Sri Lanka and Galle. In addition, information on the validated tools used in measuring PPD in Sri Lanka as stated in the previous study are also relevant to this study. They could have been stated and appropriately referenced in this section as well.*
For example, the fact that the EPDS have been translated into Sinhalese and validated in Sri Lanka is important information that should be made known in the background paper. Additionally, PPD in Sri Lanka is reported to have decreased since 2011 and the background of this paper should have captured this information.

Response: Thank you for these great suggestions. We agree that the findings from 2011 should have been included in the introduction and we have adjusted it accordingly. In the introduction we also added new information about use of cut-off scores when screening for PPD, as well as information the validated cut-off for screening in Sri Lanka and current practice.

Comment 17: *Justification of study: Lines 71-74*
The study was justified on the grounds that the similar study conducted in the same area in 2017 showed a PPD lower than that of another study conducted in Sri Lanka in 2004. However, a lot could have happened between 2004 to 2017 when the new study was conducted. Various reasons could have contributed to the difference and a proper review of the programs and interventions put in place between 2004 to 2017 could have provided more information as to why this difference was observed. Additionally, the 2017 paper indicated that there had been a general reduction in PPD from 2011, so the reduction should not really come as a surprise anyway. In my view therefore, there is not a strong justification for this study and it does not really offer anything new as it stands.

Response: We thank the reviewer for the very interesting comment. We would like to mention that the study from 2017 was published after we had collected our data in 2019 and the results were not available at the time when we made the research protocol. From your input we now realize that we should have been clearer on this issue, and not use the paper from 2017 as an argument for conducting a similar study over again as this study was not available at the time we gathered data. Nevertheless, we believe that our study provide new information about PPD in Sri Lanka as our study is the first study to use the validated cut-off 9 or more for screening for PPD in Sri Lanka. Previous

studies on PPD in Sri Lanka have used the cut-off 10 or more for reasons we are not aware of. However, the validated cut-off for screening is 9 or more and we believe that our study provides more credibility to the findings on prevalence on PPD in Sri Lanka. Furthermore, our study focuses on individual risk factors including diseases during pregnancy as well as former history of mental illness, of which the 2017 study did not provide information. For future studies on postpartum and antenatal depression in Sri Lanka it is important to use the validated cut-off for screening for postnatal depression.

Comment 18: *Recommendation: A comparative analysis or trend analysis between the previous studies from 2011 to 2017 will give a better justification for the study and provide a clearer picture of PPD in Sri Lanka. Therefore, if the data is available, I recommend that the authors provide some more information on PPD between 2011 to 2017. The authors can also make a stronger justification for the study using the timing of their administration of their EPDs if it differs significantly from other studies in Sri Lanka*

Response: Thank you for good suggestions. In our study, the mothers are screened once at clinics for PPD between 4-6 weeks following delivery, not at two different timepoints. The 2017 study screened once for postpartum depression at 10 days following delivery in Dankotuwa district and once in Galle district 4 weeks following delivery. As two different times for screening was not completed within the same population, we believe that there are several sociodemographic confounding factors that could have contributed to these results. Furthermore, when testing after 10 days the findings of PPD prevalence may be confounded by the common "postpartum blues" which is limited to 2 weeks following delivery. We think by using 4 weeks instead of the first 10 days postpartum we give a better picture of the real depressive postpartum conditions that appear.

<u>Methods</u>

Comment 19: *The collection of data using the EPD form is not really clear. Was the PPD information collected as part of the routine data collection or the team specifically went out to the field and collected this data using midwives?*
This distinction should be made clearer in the write up. It is clear that the team made use of routine records for the other variables but as things stand now one gets the impression that the PPD information was collected in the same way. I am not sure that is the case however if the previous 2017 study is anything to go by
Response: Thank you for pointing this out. We agree and have adjusted the text to be clearer.

Comment 20: *Additionally, if this is not the case then, I am also wondering why informed consent (verbal at least) was not required? Was this study simply a secondary analysis of routine data?*
Response: The EPDS and pregnancy records are routinely administered in all mothers

in Sri Lanka, meant for screening and potentially referral for further assessment and management. Therefore, as you have stated, the study is a secondary analysis of routine data. However, we understand that we were not clear on this and have adjusted the manuscript accordingly.

Comment 21: *How often is screening done for PPD at the facility?*
Response: The screening is completed once in Galle, Sri Lanka, and it is administered between 4-6 following delivery. This study digitalized the routine copies of the forms to analyze the data that is stored in the records of the clinic clients.

Comment 22: *Was the EPD form only employed because of this current study? If the screening form and PPD data is readily available, then the team could also do a trend analysis or provide some more information on PPD in the catchment area prior to the study.*
Response: Thank you for good suggestions. The EPDS is a part of a routine screening service. However, we are not in the possession of PPD data from years before, and accessing this data is not possible. Therefore, a trend analysis is unfortunately not possible to conduct.

Comment 23: *Lines 104-109: The study made use of the Tamil and Sinhala versions of the EPDS. Additionally, the study also talks about illiterate participants. Who are the illiterate participants? Are illiterate participants in this case participants who could not read Tamil and Sinhala?*
Response: Thank you for pointing this out. Agree, we have accordingly revised line 121-122 to emphasize who the illiterate participants are.

<u>Results</u>

Comment 24: *The results are well presented. Some of the confidence intervals are really wide though and the authors should take a second look at their analyses and the assumptions made for their models. Example New born death aOR 28.9 (4.5-185.1). The authors did not present any findings from their chi square analysis.*
Response: Thank you. We agree with this comment. As you know when the cases are few the confidence intervals of the estimates are wide, which is what we see with the mothers who lost their child. We prefer to give estimates and statistical significance shown as 95% confidence limits instead of p-values; however, if the editor insists, we can easily provide p-values in addition to the 95% CI.

<u>Discussion</u>

Comment 25: *Line 209: According to the authors they dealt with women who had just given birth. However, in earlier chapters, they spoke about women who had given birth 4 to 6 weeks ago. The authors should be consistent as time is important in measurement of PPD.*

The study in 2017 for example had two time points (10 days and 4 weeks). Recommendation: In the 2017 study, it was recommended that further studies on the effect of time since delivery on PPD should be looked at. Since the form is administered routinely, the authors can look at the different time periods that the forms are administered to make a stronger case about PPD in Sri Lanka.
Response: We agree and we have adjusted the text to be clearer in line 230. Regarding effect of time since delivery, please see the response to comment 18 which explains why a study on different times for screening is not possible to conduct with the data we have available.

<u>Conclusion</u>

Comment 26: *The conclusion here is much better than that the one in the abstract. The authors could look at revising the one in the abstract*
Response: Thank you. We have adjusted the conclusion in the abstract accordingly.
