# Supplementary material for: Mothers at risk of postpartum depression in Sri Lanka: A population-based study using a validated screening tool
Source: PLoS One. 2022 May 25;17(5):e0268748. doi: 10.1371/journal.pone.0268748 (PMC9132337; doi:10.1371/journal.pone.0268748)
Supplement: Attachment — Submitted filename: Response to reviewers.docx [file pone.0268748.s002.docx]

**EDITOR**

*Please ensure that your manuscript meets PLOS ONE's style requirements, including those for file naming. The PLOS ONE style templates can be found at 
https://journals.plos.org/plosone/s/file?id=wjVg/PLOSOne_formatting_sample_main_body.pdf and* 
[*https://journals.plos.org/plosone/s/file?id=ba62/PLOSOne_formatting_sample_title_authors_affiliations.pdf*](https://journals.plos.org/plosone/s/file?id=ba62/PLOSOne_formatting_sample_title_authors_affiliations.pdf)

*We note that you have indicated that data from this study are available upon request. PLOS only allows data to be available upon request if there are legal or ethical restrictions on sharing data publicly. For more information on unacceptable data access restrictions, please see http://journals.plos.org/plosone/s/data-availability#loc-unacceptable-data-access-restrictions.*

*In your revised cover letter, please address the following prompts:*

**a)** *If there are ethical or legal restrictions on sharing a de-identified data set, please explain them in detail (e.g., data contain potentially sensitive information, data are owned by a third-party organization, etc.) and who has imposed them (e.g., an ethics committee). Please also provide contact information for a data access committee, ethics committee, or other institutional body to which data requests may be sent.*

**b)** *If there are no restrictions, please upload the minimal anonymized data set necessary to replicate your study findings as either Supporting Information files or to a stable, public repository and provide us with the relevant URLs, DOIs, or accession numbers. For a list of acceptable repositories, please see* [*http://journals.plos.org/plosone/s/data-availability#loc-recommended-repositories*](http://journals.plos.org/plosone/s/data-availability#loc-recommended-repositories)*.*

**Additional Editor Comments:

Comment 1:** *The title of the current paper and that by Fan et al., 2020 are quite similar. Could you please come out clearly as to what differs between your paper and the earlier published one, more so when the study population seems to be the same*
**Response:** Thank you for pointing this out. In the revised version we have chosen to highlight that this study uses the validated cut-off for screening for PPD. The title is changed to “Risk of postpartum depression in Sri Lanka: A population-based study using a validated screening tool”.

**Comment 2:** *Lines 17 & 40: The prevalence estimates provided here were 1996 figures... could there not have been much more recent ones? Also add the estimates from South Asia - as you compare it in the conclusion*
**Response**: Good point. We have changed the reference on prevalence estimates in line 16 and line 104 to a more recent one by Hahn-Holbrook (2018). We also added prevalence ranges of PPD in South Asia in the introduction in line 73. However, we removed the sentence “this is lower than other countries in South Asia” in the conclusion as we have put more focus to screening for PPD in Sri Lanka in the revised version.

**Comment 3:** *Lines 56 - 58: I am not clear about this point... what has this to do with PPD? I read over the cited article, did not come across a relationship between poverty and quality of parenting... please clarify...*
**Response**: We have deleted this sentence.

**Comment 4:** *Lines 92 - 93: Why the assumption based on 1996 figures? More so, when there was a 2017 study in the same population?*
**Response:** At the time of planning and estimating our sample size we did not have the 2017 figures. As we knew that around 1000 births take place each calendar year in Galle, we estimated that in our sample 1000 mothers would give a reasonable precision and power. We calculated samples size both using the 1996 figures and using lower hypothetical figures with not related references. Basically our considerations tested more but only referred to the published old number. As we have now changed the 1996 reference in the introduction to a more recent one, we also updated the sample size estimation reference to be 18% Hahn-Holbrook (2018).
 **Comment 5:** *Lines 94 - 95: Why all mothers????? We the readers are not privy to information on deliveries in the study community... Also, the results section (line 159) has 975 persons... you have not clearly stated how was that arrived at... were those all the mothers interviewed? any mothers not included? response rate?*
**Response:** Thank you for pointing this out. Basically all women who attended clinic after delivery are asked to fill the EPDS questionnaire screening them for postpartum depression. It is part of the routine public services at Galle, and we do not know whether any of them refused to fill the form, as we have only the filled forms. Exclusion criteria was incomplete EPDS records. We adjusted the methods section according to you recommendation, and added a line in the first paragraph in results section in line 177 to make this clearer.

**Comment 6:** *Line 98: Will suggest this section be structured as e.g., data collection methods, tools, and process.*
**Response:** Thank you for this great suggestion. We have now made the following subsections under methods “study design and study setting”, “participants”, “study tool”, “data collection”, “outcome measures and exposure variables”, “data analysis” and “ethical issues”.

**Comment 7:** *Lines 106 - 107: check this.... specificity????*
**Response:** Thank you for notifying this mistake. It has now been corrected in line 117-118.

**Comment 8:** *Lines 109 - 115: Is the information presented here related to data collection?*
**Response:** No, the information presented was related to the current practice on screening for PPD in Sri Lanka. Thank you for notifying this. We have now divided the methods section into more subheadings to separate our data collection from the study tool according to your recommendation.

**Comment 9***: Line 114: Is the ICD used for classification or diagnosis?*
**Response:** Yes, we have now changed the line 127 to be more precise.

**Comment 10:** *Line 126: Label this as a sub-section e.g., variables considered.*
**Response:** Thank you for this recommendation, we agree to add a subsection before this paragraph and have adjusted it accordingly.

**Comment 11:** *Lines 159 - 162: Please make reference to table 2.*
**Response:** Table 2 is now referred to in the text line 178.

**Comment 12:** *Lines 193 - 195: Revise the title to be much more concise.*
**Response:** We have shortened the title of table 2.

**Comment 13:** *Line 201: Mention "EPDS Question 10" in the "Methods section" as part of the data collection tools.*
**Response**: Thank you for this recommendation. We added this information under methods section in line 118.

**Comment 14:** *Lines 214 - 216: Were these characteristics also in the 2017 study? If so, state that clearly... if not, then provide the appropriate citation.*
**Response:** Thank you, this needed clarification. The factors in our study that showed the strongest association with PPD was former history of mental illness and death of the newborn, both not mentioned in the 2017 study. Hypertension was associated with PPD and not studied separately in the former study. High maternal age was associated with PPD in the former study, and this is mentioned in maternal age section in discussion.

**Comment 15:** *Finally, please follow the reporting guidelines for Cross-sectional studies as provided in the link to guide your paper: https://www.equator-network.org/reporting-guidelines/strobe/. Also review the PLOS ONE guidelines on publications:* [*https://journals.plos.org/plosone/s/criteria-for-publication*](https://journals.plos.org/plosone/s/criteria-for-publication)
**Response:** Thank you for providing us with this information. We will adjust the paper according to the guidelines provided.

**REVIEWER**

Reviewer #1: The study addresses a key issue however there are some modifications required.
 **Background**

**Comment 16:** *The authors provide some useful information about postpartum depression in the background. Considering that a very similar study had already been conducted in the catchment area however, I think the authors could have provided more information on PPD in Sri Lanka and Galle. In addition, information on the validated tools used in measuring PPD in Sri Lanka as stated in the previous study are also relevant to this study. They could have been stated and appropriately referenced in this section as well.
For example, the fact that the EPDS have been translated into Sinhalese and validated in Sri Lanka is important information that should be made known in the background paper. Additionally, PPD in Sri Lanka is reported to have decreased since 2011 and the background of this paper should have captured this information.*

**Response:** Thank you for these great suggestions. We agree that the findings from 2011 should have been included in the introduction and we have adjusted it accordingly. In the introduction we also added new information about use of cut-off scores when screening for PPD, as well as information the validated cut-off for screening in Sri Lanka and current practice.

**Comment 17:** *Justification of study: Lines 71-74
The study was justified on the grounds that the similar study conducted in the same area in 2017 showed a PPD lower than that of another study conducted in Sri Lanka in 2004. However, a lot could have happened between 2004 to 2017 when the new study was conducted. Various reasons could have contributed to the difference and a proper review of the programs and interventions put in place between 2004 to 2017 could have provided more information as to why this difference was observed. Additionally, the 2017 paper indicated that there had been a general reduction in PPD from 2011, so the reduction should not really come as a surprise anyway. In my view therefore, there is not a strong justification for this study and it does not really offer anything new as it stands.*

**Response**: We thank the reviewer for the very interesting comment. We would like to mention that the study from 2017 was published after we had collected our data in 2019 and the results were not available at the time when we made the research protocol. From your input we now realize that we should have been clearer on this issue, and not use the paper from 2017 as an argument for conducting a similar study over again as this study was not available at the time we gathered data. Nevertheless, we believe that our study provide new information about PPD in Sri Lanka as our study is the first study to use the validated cut-off 9 or more for screening for PPD in Sri Lanka. Previous studies on PPD in Sri Lanka have used the cut-off 10 or more for reasons we are not aware of. However, the validated cut-off for screening is 9 or more and we believe that our study provides more credibility to the findings on prevalence on PPD in Sri Lanka. Furthermore, our study focuses on individual risk factors including diseases during pregnancy as well as former history of mental illness, of which the 2017 study did not provide information. For future studies on postpartum and antenatal depression in Sri Lanka it is important to use the validated cut-off for screening for postnatal depression.

**Comment 18:** *Recommendation: A comparative analysis or trend analysis between the previous studies from 2011 to 2017 will give a better justification for the study and provide a clearer picture of PPD in Sri Lanka. Therefore, if the data is available, I recommend that the authors provide some more information on PPD between 2011 to 2017. The authors can also make a stronger justification for the study using the timing of their administration of their EPDs if it differs significantly from other studies in Sri Lanka*

**Response:** Thank you for good suggestions. In our study, the mothers are screened once at clinics for PPD between 4-6 weeks following delivery, not at two different timepoints. The 2017 study screened once for postpartum depression at 10 days following delivery in Dankotuwa district and once in Galle district 4 weeks following delivery. As two different times for screening was not completed within the same population, we believe that there are several sociodemographic confounding factors that could have contributed to these results. Furthermore, when testing after 10 days the findings of PPD prevalence may be confounded by the common “postpartum blues” which is limited to 2 weeks following delivery. We think by using 4 weeks instead of the first 10 days postpartum we give a better picture of the real depressive postpartum conditions that appear.

**Methods**

**Comment 19:** *The collection of data using the EPD form is not really clear. Was the PPD information collected as part of the routine data collection or the team specifically went out to the field and collected this data using midwives?
This distinction should be made clearer in the write up. It is clear that the team made use of routine records for the other variables but as things stand now one gets the impression that the PPD information was collected in the same way. I am not sure that is the case however if the previous 2017 study is anything to go by*
**Response:** Thank you for pointing this out. We agree and have adjusted the text to be clearer.

**Comment 20:** *Additionally, if this is not the case then, I am also wondering why informed consent (verbal at least) was not required? Was this study simply a secondary analysis of routine data?*
**Response**: The EPDS and pregnancy records are routinely administered in all mothers in Sri Lanka, meant for screening and potentially referral for further assessment and management. Therefore, as you have stated, the study is a secondary analysis of routine data. However, we understand that we were not clear on this and have adjusted the manuscript accordingly.

**Comment 21:** *How often is screening done for PPD at the facility?*
**Response:** The screening is completed once in Galle, Sri Lanka, and it is administered between 4-6 following delivery. This study digitalized the routine copies of the forms to analyze the data that is stored in the records of the clinic clients.

**Comment 22:** *Was the EPD form only employed because of this current study? If the screening form and PPD data is readily available, then the team could also do a trend analysis or provide some more information on PPD in the catchment area prior to the study.*
**Response**: Thank you for good suggestions. The EPDS is a part of a routine screening service. However, we are not in the possession of PPD data from years before, and accessing this data is not possible. Therefore, a trend analysis is unfortunately not possible to conduct.

**Comment 23:** *Lines 104-109: The study made use of the Tamil and Sinhala versions of the EPDS. Additionally, the study also talks about illiterate participants. Who are the illiterate participants? Are illiterate participants in this case participants who could not read Tamil and Sinhala?*
**Response:** Thank you for pointing this out. Agree, we have accordingly revised line 121-122 to emphasize who the illiterate participants are.

**Results**

**Comment 24:** *The results are well presented. Some of the confidence intervals are really wide though and the authors should take a second look at their analyses and the assumptions made for their models. Example New born death aOR 28.9 (4.5-185.1). The authors did not present any findings from their chi square analysis.*
**Response:** Thank you. We agree with this comment. As you know when the cases are few the confidence intervals of the estimates are wide, which is what we see with the mothers who lost their child. We prefer to give estimates and statistical significance shown as 95% confidence limits instead of p-values; however, if the editor insists, we can easily provide p-values in addition to the 95% CI.

**Discussion**

**Comment 25:** *Line 209: According to the authors they dealt with women who had just given birth. However, in earlier chapters, they spoke about women who had given birth 4 to 6 weeks ago. The authors should be consistent as time is important in measurement of PPD. The study in 2017 for example had two time points (10 days and 4 weeks).* *Recommendation: In the 2017 study, it was recommended that further studies on the effect of time since delivery on PPD should be looked at. Since the form is administered routinely, the authors can look at the different time periods that the forms are administered to make a stronger case about PPD in Sri Lanka.*
**Response**: We agree and we have adjusted the text to be clearer in line 230. Regarding effect of time since delivery, please see the response to comment 18 which explains why a study on different times for screening is not possible to conduct with the data we have available.

**Conclusion**

**Comment 26:** *The conclusion here is much better than that the one in the abstract. The authors could look at revising the one in the abstract*
**Response:** Thank you. We have adjusted the conclusion in the abstract accordingly.
