# Supplementary material for: Mothers at risk of postpartum depression in Sri Lanka: A population-based study using a validated screening tool
Source: PLoS One. 2022 May 25;17(5):e0268748. doi: 10.1371/journal.pone.0268748 (PMC9132337; doi:10.1371/journal.pone.0268748)
Supplement: Attachment — Submitted filename: Response to reviewers.docx [file pone.0268748.s004.docx]

**General comment**
We have now corrected grammatical errors and sentence structure in numerous sections. Furthermore, we added a paragraph in the introduction about thoughts of self-harm, and moved the self-harm paragraph in the discussion to be after the paragraph about newborn death and former history of mental illness as risk factors for EPDS score 9 or more (risk of PPD). We also added a new reference to the reference list in line 441.

**Additional Editor Comments**

**Comment 1**: *Though the title has been revised, the content of the paper seems to be at variance. Is the focus on "Risk of PPD", OR "Prevalence of PPD" or "Prevalence of risk of PPD"? That should come out clearly and consistently throughout the manuscript.*
**Response:** Thank you for this comment. Our focus is the prevalence of mothers at risk of PPD (as measured by mothers with an EPDS score 9 or more). We have now edited the manuscript and title to be consistent in terminology.

**ABSTRACT**
**Comment 2:** *Lines 40 & 41: Please revise the grammar and sentence structure to enhance clarity.*
**Response:** Thank you for pointing this out, we have changed the sentence in line 22-24 to make it clearer.

**Comment 3:** *Line 42: Should the sentence not be "The prevalence of PPD among mothers with EPDS score 9 and above was..."? To the best of my understanding, the prevalence is of PPD and not EPDS.*
**Response:** Thank you for raising this issue. The EPDS is a screening tool to identify mothers at risk of PPD, but a final diagnosis is made through clinical interview with the mothers identified. Therefore, we believe that the sentence “mothers with an EPDS score 9 or more (risk of PPD)” is more precise than stating “mothers with PPD”; and we have adjusted the terminology throughout the manuscript to be consistent on this matter; in the risk factor sections we have changed the wording to “risk of EPDS score 9 or more”. As previous studies in Sri Lanka have used a higher cut-off for screening for PPD, the prevalence estimates cannot be compared correctly without stating which EPDS score is being used.

However, we can use the terminology “prevalence of PPD” consistently throughout the paper if Editor prefers, but we believe that this terminology is less precise.

**Comment 4:** *Line 47: The "thoughts of self-harm" should be appropriately linked to PPD... Just mentioning it does not add any value to its presence...*
**Response:** Thank you, this needed clarification. We have added a new paragraph in the introduction, to describe the relationship between serf-harm and PPD in line 86-92.

**Comment 5*:*** *Line 48: can 9.4% be referred to as "a significant proportion"???*
**Response:** We have adjusted the line 39.

**Comment 6:** *Lines 50 & 51: Would it not have been simpler to just say as the data shows that "a higher cut-off produces reduced PPD prevalence estimates"? The current statement is quite ambiguous...*
**Response:** We agree to this comment, and have adjusted the line 41-44.

**INTRODUCTION**
**Comment 7:** *Line 64: "suggest" or "suggests"?* **Response:** We have corrected the line 56.

**Comment 8:** *Lines 73 & 74: how is this related to PPD? that would be very helpful to contextualize the information on "self-harm".*
**Response:** Thank you for pointing this out. We have added a paragraph in line 86-92 to make this more clear.

**Comment 9:** *Line 94: "put focus" does not seem to be the right expression*
**Response:** We agree to this point and have adjusted the sentence in line 80.

**Comment 10:** *Line 98, 104 & 105: Revise the sentences grammatically and structurally to enhance its clarity*
**Response:** The sentences has been adjusted in line 95 and line 102-103.

**Comment 11:** *Lines 106: Is the focus of the study "Prevalence of mothers at risk of PPD" or "prevalence of mothers with PPD"????*
**Response:** Thank you for addressing the inconsistent use of terminology. As stated in comment 3, the focus in our paper is mothers at risk of PPD defined as mothers with an EPDS score 9 or more and we have adjusted the manuscript and title to be consistent.

**Comment 12:** *Line 113: Under point 1, which study is being "repeated"?*
**Response:** We have changed the sentence in line 107-108.

**Comment 13:** Line 114: Postpartum women do not attend "antenatal clinics", so please revise
**Response:** We have changed the line 107-108.

**PARTICIPANTS**
**Comment 14:** *Line 138: Would it not be best to add that this sample size (907) was close to the 975 that delivered over the period and as such they were all included in the study?*
**Response:** Thank you for this comment. However, as the sample size estimation was completed before we had the actual number of births in 2019, we believe that our sentence describes this more clearly.

**Comment 15:** *Line 140 & 141: Why not say "mothers with incomplete EPDS records were excluded from the study"?*
**Response:** We have adjusted the sentence in line 133-134 accordingly. **STUDY TOOL**
**Comment 16:** *Line 152 & 153: The sentence seems to be contained in the next, so why not incorporate one into the other?*
**Response:** We incorporated the sentences in line 141-144.

**Comment 17:** *Line 156: Would be great if the influence of "thoughts of self-harm" on PPD could be clearly stated*
**Response:** We added a paragraph in the introduction to describe the relationship between PPD and self-harm. We deleted the sentence in line 145.

**DATA COLLECTION**
**Comment 18:** *Line 170: pregnant women in this study cannot have EPDS records as the information is collected after 4 weeks of delivery.*
**Response:** Good point, we have changed the line 158 to be more precise.

**DATA ANALYSIS**
**Comment 19**: *Lines 199 - 202: The non-use of the Pearson Chi-square test in data analysis was raised by Reviewer 1.... this was not reflected in the results section...*
**Response:** As we have not used the results of the Pearson Chi-square in the tables, we have now deleted the sentence in line 186-188.

**Comment 20:** *Lines 206 - 208: "Thoughts of self-harm" seem to be a very important feature of this study - could you clearly state the importance of this to PPD or its influence on the study outcomes clearly?*
**Response:** We have inserted a paragraph in the introduction to describe the importance of self-harm thoughts in PPD and have adjusted the discussion in line 353-355 to be more precise.

**RESULTS**
**Comment 21:** *Line 218: The study was not about "pregnant women", so please revise*
**Response:** We have adjusted the line 205.

**DISCUSSION**
**Comment 22:** *Line 276: which is the former publication??? any citations?*
**Response:** Citation has been added in line 270.

**Comment 23:** *Line 281: "Newborn death" is not a maternal characteristic*
**Response:** We agree to this comment and have adjusted the line 272-273.

**Comment 24:** *Line 286: What are you implying with the statement "our material"???*
**Response:** We have changed the wording in line 276-278.

**Comment 25:** *Line 293: The statement is contrary to that of the first sentence in the conclusion of the abstract...*
**Response:** The conclusion has been changed in line 39-40.

**Comment 26:** *Line 299: Check your grammar*
**Response:** It has been corrected in line 292.

**Comment 27*:*** *Line 360: the available "study population" was used... why not state that instead of "adequate sample size"?*
**Response:** We have adjusted the sentence in line 372.

**Comment 28:** *Line 366: is it "prevalence of PPD" or "risk of PPD" or "prevalence of risk of PPD"???? Please be consistent all through the manuscript...*
**Response:** Thank you for pointing this out. We have adjusted the manuscript to make this clearer in numerous sections.

**CONCLUSION**
**Comment 29:** *Line 381: Should the initial part of the sentence not be "the prevalence of PPD" or "risk of PPD at an EPDS score of 9 or more"???? Thought it was the "prevalence of PPD being measured with the EPDS" and "not the prevalence of EPDS".*
**Response:** We have adjusted the sentence in line 394-396 to be more precise.

**Comment 30:** *Line 384: In place of "had a substantial impact", why not state clearly that "there was a drop in prevalence rates with higher cut-offs for EPDS"??? That would be much more to the point*
**Response:** We have rephrased the sentence in line 397-399 accordingly.
